# Distinct kinematics and micromorphology for symmetrical rowing and sliding on water in ripple bugs and water striders

**DOI:** 10.1038/s41598-025-28453-0

**Published:** 2025-12-29

**Authors:** Sang Yun Bang, Woojoo Kim, Jeongseop Lee, Jinseok Park, Versha Khare, Sang-im Lee, Piotr Grzegorz Jablonski

**Affiliations:** 1 School of Biological Sciences, Seoul National University, Seoul, South Korea; 2 Insitutite of Biodiversity, Seoul National University, Seoul, South Korea; 3 Research Institute of Basic Sciences, Seoul National University, Seoul, South Korea; 4 Soft Foundry Institute, College of Engineering, Seoul National University, Seoul, Republic of Korea; 5 Laboratory of Integrative Animal Ecology, Department of New Biology, DGIST, Daegu, South Korea; 6 Museum and Institute of Zoology, Polish Academy of Sciences, Warsaw, Poland

**Keywords:** Behavioural ecology, Animal behaviour, Biomechanics, Entomology

## Abstract

Semiaquatic bugs evolved two different propulsion mechanisms for their symmetrical rowing: a drag-based propulsion in Veliidae and a surface-tension-based propulsion in Gerridae. However, the comparative leg micromorphology and kinematics underlying these two mechanisms remain underexplored. In this study, we compared leg micro- and nanostructures and kinematics of *Rhagovelia distincta* (Veliidae), which employs midleg fans as oar-like blades for drag-based thrust, with *Gerris latiabdominis* (Gerridae), which uses long midlegs for surface-tension-based thrust. *R. distincta* move their midlegs in short strokes and deployed fans which function as “leaky paddles” with higher anteroposterior rigidity, inferred from seta and setula structure, exploiting drag and potentially lift. Fan protraction into the water appeared to require muscle control, while elastocapillarity may contribute to fan shaping. In contrast, *G. latiabdominis* exhibited longer strokes with midlegs covered with dense hydrophobic hairs suited for surface-tension-based propulsion. Ventral setae on tarsal section producing surface-tension-based-thrust formed longitudinal rows-and-gaps in both species, with posterior rows particularly robust and nano-grooved in *G. latiabdominis*. Additionally, both formed ventral beam-like structures from overlapping flat-tipped setae on hindlegs and forelegs which are used for support and sliding. These findings generate new hypotheses for refining models of locomotion on water surface by insects with their micro/nano-morphological diversity.

**Supplementary Information:**

The online version contains supplementary material available at 10.1038/s41598-025-28453-0.

## Introduction

Locomotion on the water surface presents a unique set of physical challenges that have driven repeated and diverse evolutionary solutions among insects^1^. The semiaquatic bugs (Gerromorpha) provide an example of adaptive radiation into this novel environment. These insects evolved distinct morphological, behavioral, and anatomical traits that enable movement on the air-water interface^2^. These adaptations reflect multiple, lineage-specific strategies for solving similar functional problems–offering a model system for studying how alternative solutions involving morphology and behavior evolve under ecological constraints of physical environment–the water surface. However, the details of the co-evolutionary and functional interplay between leg microstructures, stroke kinematics, and thrust mechanics across major independent lineages of this adaptive radiation remain poorly understood. To provide more insights into this question, we compare two major independently evolved solutions for symmetrical rowing in Gerromorpha. Here, we focus on how alternative physical mechanisms–drag-based versus surface-tension-based thrust–are realized through contrasting yet functionally convergent morphologies.

Notably, two lineages—Veliidae (a polyphyletic family typically found in fast-flowing streams) and Gerridae (typically inhabiting slow or still waters)—have independently evolved symmetrical backward rowing by midlegs for forward thrust^2–4^. While both rely on midlegs for thrust and fore- and hindlegs for support and sliding, their physical modes of thrust generation differ: Veliidae exploit hydrodynamic drag (and potentially lift), while Gerridae generate thrust primarily through surface tension forces^5,6^. Despite their independent origins and these contrasting mechanisms, the leg kinematics and microstructural adaptations that support these behaviors have not been systematically compared.

Within Veliidae, species such as those in the genus *Rhagovelia* possess specialized midleg pretarsal structures known as swimming fans, which function as oars^2,5,7–9^. These structures are assumed to be actively controlled via a claw retractor muscle^5^, though recent observations of isolated fans spreading in water have led to the hypothesis of passive elasto-capillary spreading^8,9^. The nature of fan manipulation in intact, behaving animals remains unresolved. Similarly, the ventral microstructures on midlegs (involved in thrust) and on fore- and hindlegs (involved in support and sliding) have not been systematically examined in *Rhagovelia*, though their role in generating surface tension-based forces via water dimples is likely.

In contrast, Gerridae species such as *Gerris latiabdominis* do not possess swimming fans but instead have elongated, hairy midlegs that generate thrust through the creation of asymmetric dimples on the water surface. These midlegs, along with highly hydrophobic ventral surfaces, are critical for surface-tension-based propulsion^6^. While some aspects of locomotory performance have been compared^3^, detailed kinematic analyses and high-resolution comparisons of leg micromorphology between Gerridae and Veliidae are lacking. Prior studies have presented images of leg hair arrangements^2,10^, but a focused comparative analysis across functional leg regions has not been conducted since Andersen (1976).

Following the framework set by Andersen (1976, 1982) and Crumière et al. (2016), we compare *Rhagovelia distincta* (Veliidae) and *Gerris latiabdominis* (Gerridae), two small-bodied representatives of their respective clades. We examine their midleg microstructures and stroke kinematics to evaluate how different physical thrust mechanisms are supported by contrasting anatomical features. We further investigate the hypothesized passive versus active mechanisms of fan control in *R. distincta*. Finally, we describe and compare fore- and hindleg microstructures used in support and sliding to identify potential convergences across taxa with different thrust mechanisms. Our results provide a foundation for future work on the functional evolution of water-surface locomotion in Gerromorpha.

## Results

### Overview of figures and supplementary materials

**Fig. 1:**
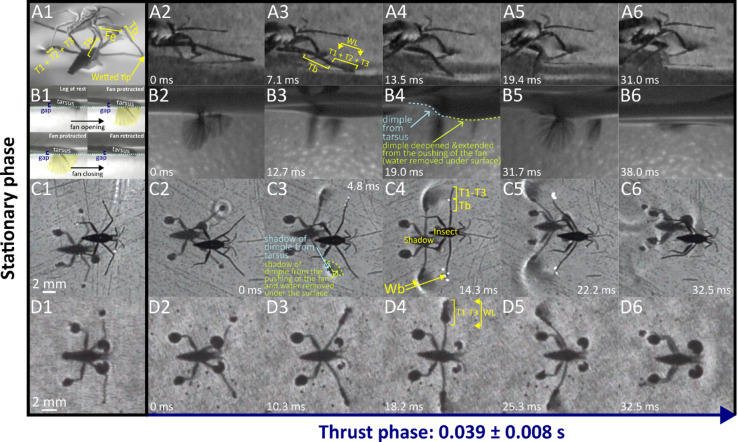
*Rhagovelia distincta* during the thrust phase. (A1–A6) Side view above the water surface, showing interactions between the leg and water surface. (B1–B6) Side view below the surface, highlighting the motion of the swimming fan during the thrust phase of a stroke; (B1) Examples of fan opening and closing that are not associated with changes in gap size between water surface and distal surface illustrating how fan protracts and retracts without changes of tarsal position relative to water surface (SI Part 2; Figures S4 and S5). (C1–C6) Top view capturing body and leg positions throughout the thrust phase. (D1–D6) Bottom view from beneath the container, showing shadows cast by the body and water-surface dimples. Abbreviations: Fe – femur; Tb – tibia; T1–T3 – tarsomeres 1–3; WL – wetted midleg length; Wb – wave bow. Panels B4 and C3: blue and green annotations indicate interpreted differences in dimple shape based on comparisons with *G. latiabdominis* (see Fig. 2 and Figure S2).

**Fig. 2:**
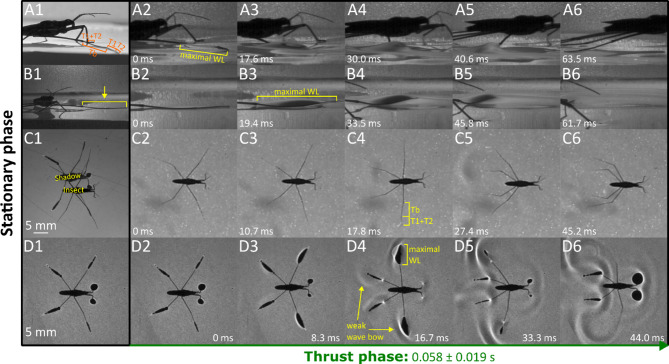
*Gerris latiabdominis* during the thrust phase. (A1–A6) Side view above the water surface, showing midleg motion during thrust. (B1–B6) Side view above the water, focused on midleg interaction with the water surface. (C1–C6) Top view capturing body and leg positions throughout the thrust phase. (D1–D6) Bottom view from beneath the container, showing shadows cast by the body and water-surface dimples. Abbreviations: Tb – tibia; T1–T2 – tarsomeres 1 and 2; WL – wetted midleg length; Wb – wave bow.

**Fig. 3:**
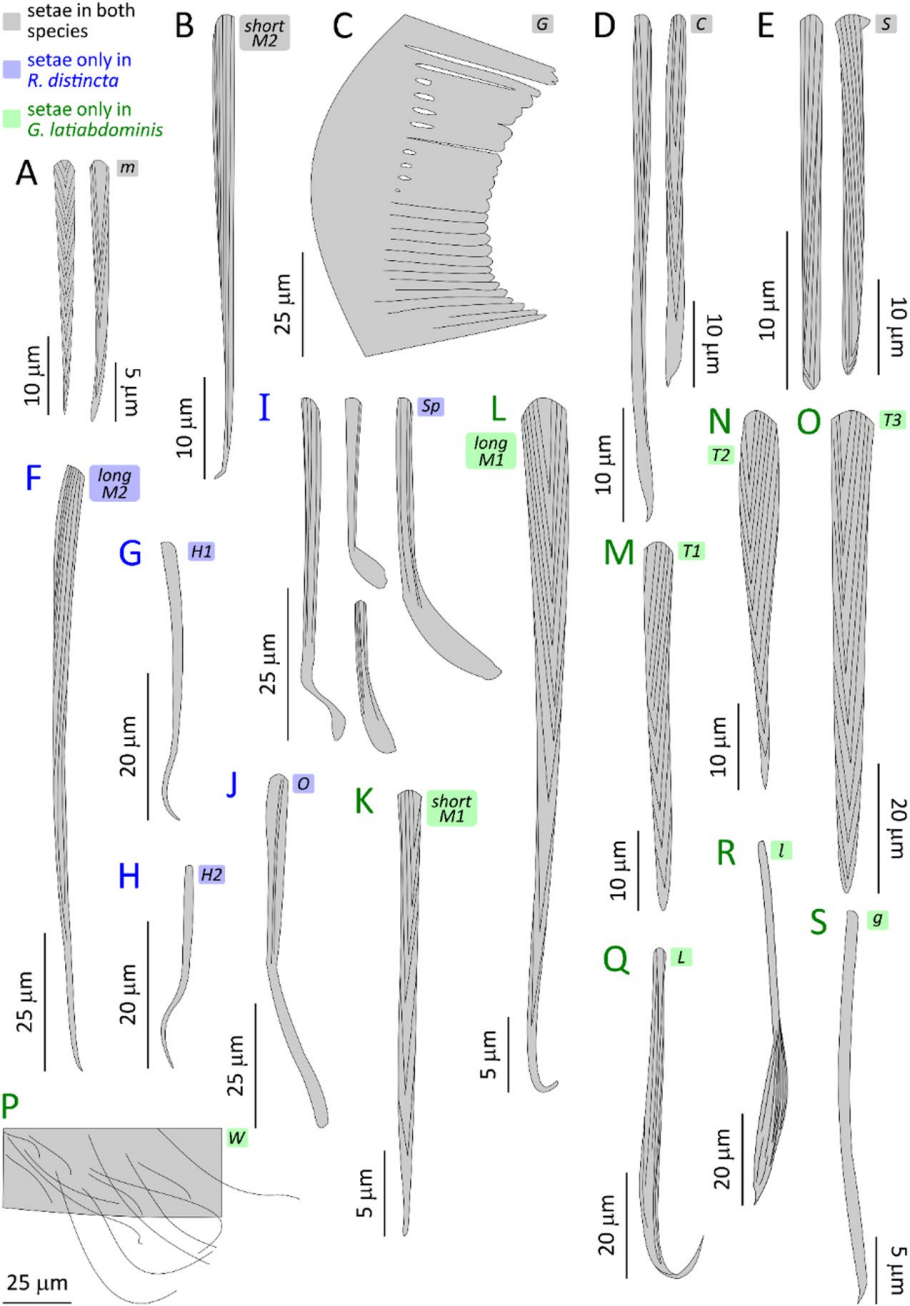
Schematic drawings of different types of setae found on leg sections of *Rhagovelia distincta* and *Gerris latiabdominis* that interact with the water surface. (**A**) Microsetae, *m*. (**B**) Macrosetae 2, *M2*. (**C**) Grooming comb, *G*. (**D**) Cuspidate setae, *C*. (**E**) Stumped setae, *S*. (**F**) Macrosetae 2, *M2*. (**G**) Hook setae 1, *H1.* (**H**) Hook setae 2, *H2*. (**I**) Spoon setae, *Sp*. (**J**) Obtuse setae, *O*. (**K–L**) Macrosetae 1, *M1*. (**M**) Thorn setae 1, *T1*. (**N**) Thorn setae 2, *T2*. (**O**) Thorn setae 3, *T3*. (**P**) Web setae, *W*. (**Q**) Leaf-blade setae, *L*. (**R**) Leaf-like setae, *l*. (**S**) Grass-blade setae, *g*. Alphabetical labels correspond to SEM photos in Figure S10 and morphological data in Table S3. Setae found in both species are labeled in **black**; those found only in *R. distincta* are in **blue**; and those found only in *G. latiabdominis* are in **green**. Note that scales vary between panels and are specified for each seta type. Descriptions of all seta types are provided in Supplementary Information Part 3 C.

**Fig. 4:**
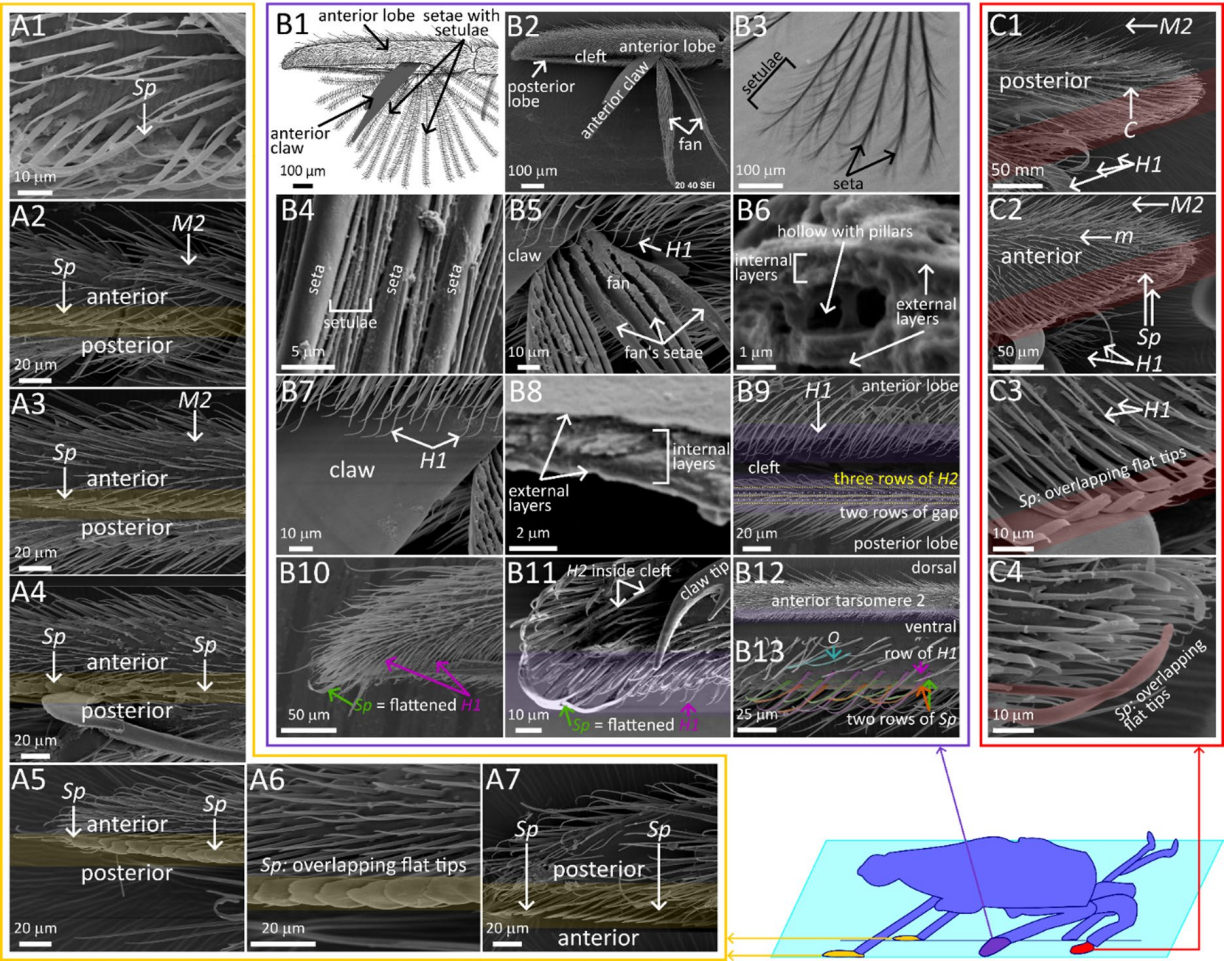
Scanning electron microscopy summary of leg microstructures in *Rhagovelia distincta*. (A) Hindleg: (A1) a row of spoon setae, *Sp*, on proximal ventral tarsomere 2; (A2) anterio-ventral view of the tarsal joint (between tarsomeres 1 and 2), showing overlapping *Sp* setae with flattened tips, flanked by macrosetae 2, *M2*; (A3–A5) anterio-ventral views from proximal tarsomere 2 to the tarsal tip, showing a progressively beam-like structure formed by overlapping *Sp* setae on the ventral side; (A6) a row of spoon setae, *Sp*, on distal tarsomere 2; (A7) posterior-lateral view of distal tarsomere 2 with a row of spoon setae, *Sp*, along the water-interacting ventral side (yellow shading in A2–A7). (B) Midleg: (B1) schematic of the pretarsal swimming fan used for hydrodynamics-based thrust; (B2) anterio-ventral view of tarsomere 3 showing the fan and anterior claw extending from the cleft between two lobes (posterior claw not visible); (B3) protracted fan in water showing hierarchical structure of setae and setulae; (B4) anterior view of folded fan, highlighting relative thickness of setae and setulae; (B5) “board-like” cross-sectional shape of fan; (B6) cross-section of a fan seta showing internal layers, hollow core with pillars, and outer layers; (B7) surface of the anterior claw extruding from the cleft surrounded by hook setae (anterior lobe`s *H1* setae visible); (B8) cross-section of the claw; (B9) Ventral edges of the cleft with rows of *H1* (anterior lobe) and *H2* (posterior lobe) setae; (B10) anterior view of the distal portion of tarsomere 3; (B11) close-up of the anterior lobe tip showing long, flattened modified *H1* setae resembling spoon setae, *Sp*, and internal cleft wall lined with *H2*; tip of anterior claw also visible (posterior lobe removed); (B12) lateral view of tarsomere 2; (B13) ventral view of tarsomere 2 showing orderly rows of *Sp* and *H1* setae. (C) Foreleg: (C1) posterior view showing sparse cuspidate setae, *C*, dorsal macrosetae 2, *M2*, and ventral hook setae, *H1*; (C2) anterio-ventral view showing microsetae, *m*, ventral hook setae, *H1*, and a row of spoon setae, *Sp* along the ventral water-interacting surface; (C3) close-up of spoon setae, *Sp*, near the claw base; (C4) array of spoon setae, *Sp*, at the tarsal tip. Color-shaded regions in SEM panels denote ventral (water-interacting) leg surfaces. Additional SEMs are provided for hindlegs in Figure S19, midlegs in Figures S11–S17, and forelegs in Figure S19.

The results are presented through figures cited in sequential order for clarity and traceability. Figure 1A1–D6 portrays typical thrust phase in *Rhagovelia distincta*, followed by corresponding views for *Gerris latiabdominis* in Fig. 2A1–D6. Figure 3A–S illustrates leg seta types. Figures 4A1–C4 and 5A1–C4 show SEMs of leg microstructures. Figure 6A–E compares contact angles and droplet shapes. Stroke kinematics and interspecific comparisons are shown in Figs. 7A–J and 8A1–D, with measurement summaries in Fig. 9A–H.

Supplementary figures follow the same order. Figure S1A–B depicts resting leg posture on water. Figure S2A–H shows leg-surface interaction and dimple formation. Figure S3A–E quantifies fan geometry and angles. Figures S4A–D and S5 capture fan dynamics during strokes. Figures S6A–G and S7A–C detail fan positioning within the tarsal cleft. Figures S8A–E and S9A–E present dissected fan–claw complexes. Figure S10A–S expands seta classification. Figures S11, S12A–H, S13–F, S14A–F describe ultrastructural features of fan setae and claws. Figures S15A–F, S16A–D, and S17A–B highlight ventral hook and spoon setae. Figures S18A–D and S19A–H show fore- and hindleg structures in *R. distincta*. Figures S20A–F and S21A–C provide analogous views for *G. latiabdominis*. Figures S22A–D and S23A–E show additional fore- and hindleg adaptations. Figure S24A–E and (a–e) detail droplet behavior and surface wetting (contact angles). Figures S25A–D summarize net force estimates during strokes. Figures S26A–D and S27 present multivariate and theoretical analyses of kinematics and fan leakiness.

### Behavioral observations: leg use at rest

*R. distincta* body is supported on foreleg and hindleg tarsus with minimal contribution from the midleg tarsal tips (Fig. 1A1, B1, C1, D1; Figure S1). Occasionally, a very small portion of the midleg`s fan is protruded from the tarsal tip into the water body through the surface it`s in contact with (Fig. 1B1; Video S3 Part 4). *G. latiabdominis* body is supported on foreleg tarsus, hindleg tibia and tarsus, and midleg intermediate-distal tibia and tarsus (Fig. 2A1, B1, C1, D1; Video S2), which create dimples without piercing the water surface (dimples cast shadows on the bottom of the container; Fig. 2C1, D1; Video S2 Part 4).

### Behavioral observations: leg use at locomotion

During a typical initial thrust phase (Fig. 1), *R.* distincta moves its midlegs forward–either above the water or lightly contacting the surface–then places the tarsi onto the water (Fig. 1C2), which is associated with fan extension into the water (Fig. 1B2). Alternatively, the midlegs may advance while the tarsal tips remain in contact with the surface and a small portion of the fan protrudes underwater (Fig. 1B6; Figure S2D; Video S3 Part 4). Observations from 110 slow-motion videos (each capturing 1–4 fan opening and closing events) suggest that *R. distincta* actively controls the timing, extent, and duration of fan protraction and retraction, regardless of midleg position on the water surface (SI Part 2; Figure S4; Video S3). For example, we frequently observed the fan opening and closing without any respective decrease or increase in the gap between the tarsus and the water surface (Fig. 1B1). This disagrees with the recently proposed passive fan actuation hypothesis^9^, which posits that lowering the tarsus onto the water surface is required to initiate fan unfolding through fan-water elastocapillary interactions (SI Part 2).

As the fan rapidly protracts (Fig. 1B2; Figure S2C, D) and the midlegs are pushed backward (Fig. 1C4, C5), the tarsus is simultaneously pressed downward (Fig. 1C3; Figures S2B and S5). This generates growing anteroposterior asymmetrical dimples (Fig. 1B4), visible as shadows that expand from the initial miniscule circles at the tarsal tips to ovals extending distally from the tibiotarsal joints (Fig. 1D4). Contrast to *G. latiabdominis* (Fig. 2D), *R. distincta* shows an expanded anterior dimple region (Fig. 1C3), likely caused by water displaced beneath the surface by the fan (Fig. 1B4; Figure S2G, H). Strong strokes can produce surface waves (Fig. 1C4, C5; 17 of 99 strokes; Video S1 Part 1), but all strokes transition into a passive sliding phase, during which the midlegs either disengage from the surface or trail behind with minimal fan protrusion (Fig. 1B6; Video S1). Observed midleg disengagement suggests adhesive forces and surface tension are overcome in this process (Video S3). Throughout, the fore- and hindleg tarsi remain in contact with the surface, providing support during sliding.

Duration of fan protraction ranged between 6 and 23 ms (12.3 ± 3.4 ms; *n* = 69) while the duration of fan retraction ranged between 3 and 15 ms (8.4 ± 2.6 ms; *n* = 73) (Figures S3 and S4). Shorter durations were often associated with strokes beginning or ending with partially protracted fans, where 2–4 distal setae tips protruded into the water. Fully protracted fans had an average projected area of 0.89 ± 0.04 mm^2^, radius of 0.85 ± 0.01 mm, and protracted angle of 139.13 ± 3.64° (*n* = 6; Figure S3A). The longitudinal axis of wetted midleg and protracted fan typically lay in the same plane, slanted at 80.2 ± 6.0° (*n* = 26; Figure S3E).

*G. latiabdominis* generated thrust without piercing the surface (Fig. 2), using midlegs slightly rotated so that anteroventral gap-row microstructures pressed backward against the water (Figure S2; 27–36 ms into the stroke). Backward movement of the midlegs produced anteroposterior asymmetrical dimples and backward-moving surface waves (Fig. 2D; Figure S2E, F; Video S2). Midleg disengagement occurred when the wetted midleg aligned nearly parallel to the direction of body movement and proceeded gradually from proximal to distal segments. Some instances of disengagement appeared smooth (Video S2), while others suggested adhesive forces and surface tension were overcome during the process (Video S2; Figure S2E, 95 ms). Hindlegs contributed weakly to thrust, as indicated by faintly asymmetrical shadows and small wave bows during the initial stroke phase (Fig. 2D4). Hindleg tarsi and tibiae provided support during sliding, shown by shadows aligned with the body axis (Fig. 2D5, D6). Forelegs also supported the body, particularly near the end of the stroke, as indicated by prominent shadows (Fig. 2D6), except at mid-stroke when support was reduced (Fig. 2D4).

## Ventral microstructures on legs

### Overview

The legs of *R. distincta* had a less dense hair layer than those of *G. latiabdominis*. We identified 17 setae (hair) types: five shared by both species, five unique to *R. distincta*, and seven unique to *G. latiabdominis* (Fig. 3; Figure S10; Table S3). Our analysis focused on the ventral microstructures of leg segments that interact with the water surface, and respective nanometer sized details specifically in *R. distincta* (Figs. 4 and 5; Figures S11–S23).

### Ventral microstructures for thrust generation

The swimming fan of *R. distincta* (Fig. 4B1, B2; Figures S6–S9 and S11–S14) consists of anterior and posterior claws and a fan made up of 17 (Figures S8, S9) to 21 (Fig. 4B2; Figure S11) setae. Each seta bears setulae along its axis at 8–12 μm intervals, forming a feather-like structure (Fig. 4B3, B4; Figures S12, S13). When protracted, the distance between adjacent setae ranges from ~ 20 to ~ 100 μm, and between setulae from several to ~ 20 μm, with typical setula spacing of 4–10 μm (Figure S13A, B). The fan is anchored at the inner proximal corner of the cleft between the two lobes of tarsomere 3 (Fig. 4B2). The surfaces of the setae, setulae, and claws lack nanogrooves (Figure S13C–F). The setae resemble flat beams or boards (Fig. 4B5; Figure S12C) with near-elliptical (Fig. 4B6; Figure S12D) or slightly triangular (Figure S12E) cross-sections, measuring 2–4 μm × 7–10 μm. The orientation of the narrow edges suggests that they face the water during thrust generation (Fig. 4B5; Figures S12, S13). The setulae are also flat, 1–2 μm wide and 300–700 nm thick, with a hollow center (Figure S12H), and their orientation further suggest that they press against the water with the narrow edges. Transverse sections of fan setae show lamellar outer layers and a central hollow (~ 700 nm in diameter) containing internal rods (~ 400 nm in diameter) (Fig. 4B6; Figure S12). Claw cross-sections are 1.5–2.5 μm thick and consist of external lamellar layers and multiple internal layers with nanofibers (100 nm) and cluster of nanofibers (200–400 nm) running in various directions (Fig. 4B8; Figure S14).

Passive elastocapillary expansion of the fan in water (Figures S8 and S9) was observed only when the fan was completely dissected–either with or without the anterior claw–and removed from its natural position in the cleft (SI Part 3B). In contrast, observations of intact fans and claws anchored naturally within the cleft indicate that fan expansion into the water at the onset of use is not passive (Videos S1 and S3; SI Part 3B). Moreover, we were able to induce fan protraction by mechanically pulling the *ut* tendon connected to the base of the fan-and-claw structure (Figures S8 and S9), supporting the involvement of active muscular control.

**Fig. 5:**
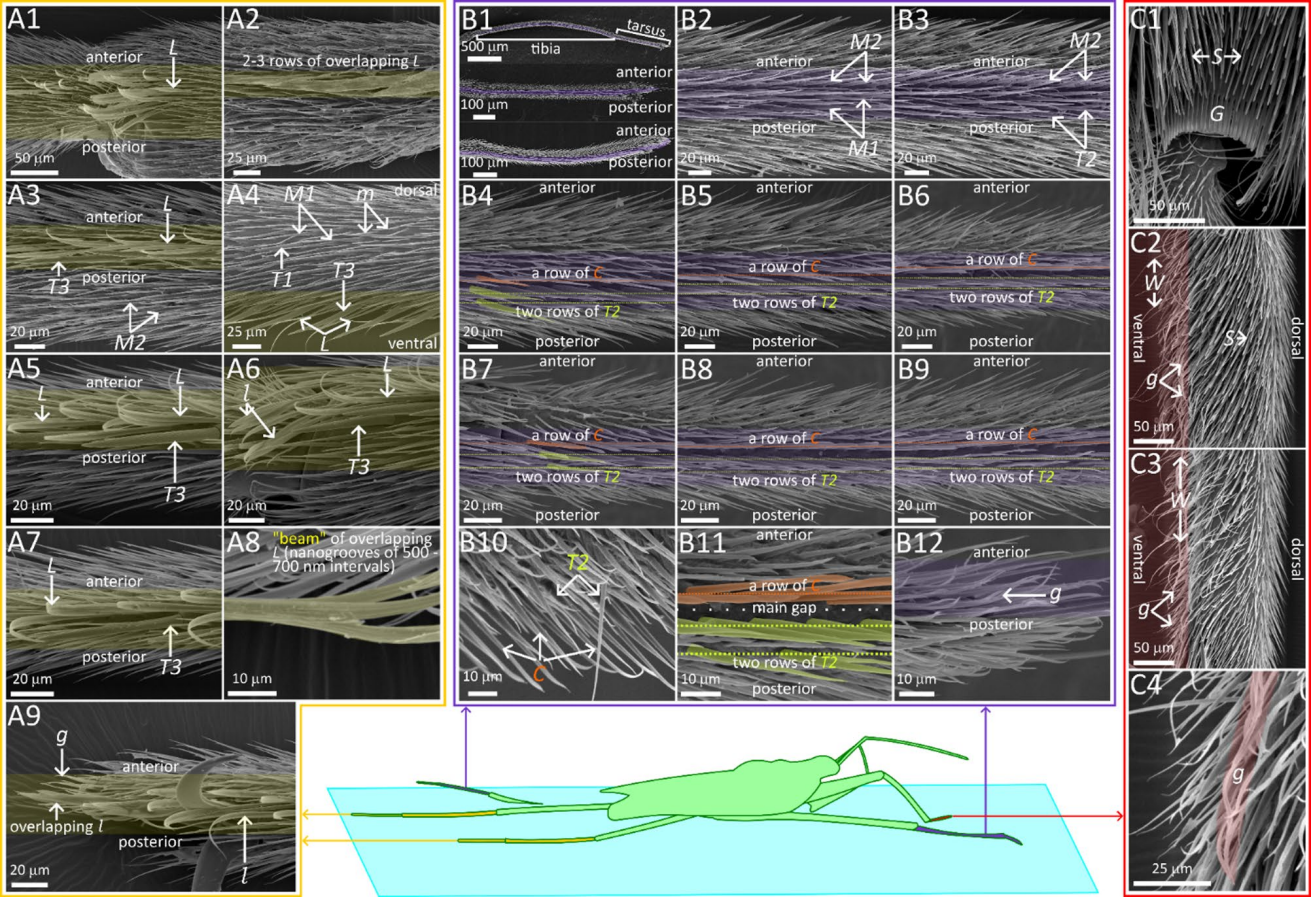
Scanning electron microscopy summary of leg microstructures in *Gerris latiabdominis*. (A) Hindleg: (A1) ventral view of femorotibial joint covered with leaf-blade setae, *L*; (A2) ventral proximal tibia showing overlapping distal sections of *L* setae forming a “beam-like” structure believed to support the insect on water; (A3) same as A2, but from a different preparation; *L* setae appear more randomly bent due to cleaning and drying procedures. A row of large thorn setae 3, *T3*, runs posterior to the *L* setae, with macrosetae 2, *M2*, present on the leg`s posterior side; (A4) lateral view of distal tibia with dense microsetae, *m*, macrosetae 1, *M1*, thorn setae 1, *T1*, and a ventral row of thorn setae 3, *T3*, on the water-interacting surface; (A5) ventral view of tarsomere 1 with a longitudinal row of leaf-blade setae, *L*, and an adjacent row of thorn setae 3, *T3*; (A6) ventral view of tibiotarsal joint with arrays of leaf-like setae, *l*, and thorn setae 3, *T3*, extending from distal tibia; (A7) ventral view of tarsomere 2 with a continuing row of leaf-blade setae, *L*, and an adjacent row of thorn setae 3, *T3.* (A8) close-up of ventral overlapping *L* setae forming a flat “beam-like” surface with nano-grooves running longitudinally; (A9) ventral view of tarsal tip with an array of leaf-like setae, *l*. (B) Midleg: (B1) overview of the “gaps and rows” arrangement on ventral midleg segments involved in thrust generation (purple shading). Top panel: full tibia and tarsus; middle panel: tarsus with one main longitudinal gap and two less distinct gaps; bottom panel: clearer visualization of the three gaps, each flanked by linear setal rows. (B2) ventral view of intermediate tibia with macrosetae 2, *M2*, flanked by rows of macrosetae 1, *M1*, separated by noticeable gaps; (B3) distal tibia with continuing macrosetae 2, *M2*, and adjacent rows of thorn setae 2, *T2*, which resemble *M1*; (B4–B6) anteroventral view of tarsomere 1, with a row of cuspidate setae, *C*, adjacent rows of thorn setae 2, *T2*, and a gap in between; (B7–B9) ventral view of tarsomere 2 with a continuing row of cuspidate setae, *C*, and two posterior rows of thorn setae 2, *T2;* visible are the main gap between *C* and *T2*, and a narrow gap between the two *T2* rows; (B10) anterior view of tarsomere 2 (B11) clear view of the main gap between *C* and *T2* rows; (B12) tarsal tip with an array of grass-blade setae, *g*. (C) Foreleg: (C1) grooming comb, *G*, and stumped setae, *S*, on dorsal tibiotarsal joint; (C2) anterior-lateral view of proximal tarsus with stumped setae, *S*, on lateral side, and overlapping grass-blade, *g*, and web setae, *W*, on the ventral water-interacting surface; (C3) posterior-lateral view of distal tarsus with grass-blade, *g*, and web setae, *W*; (C4) close-up of overlapping distal tip of grass-blade, *g*. Color-shaded regions in SEM panels denote ventral (water-interacting) leg surfaces. Additional SEMs are provided for hindlegs in Figure S23, midlegs in Figures S20 and S21, and forelegs in Figure S22.

Along the ventral edge of the posterior lobe (Figures S15A–F and S16A–D), a structure composed of three rows of *H2* setae—spaced 6–8 μm apart within each row and separated by two 2.5–5 μm wide gaps—forms a band that presses against the water surface without breaking it during a stroke (Fig. 4B9; Figure S16). This pattern resembles the “gaps and rows” arrangement seen on the tarsus of *G. latiabdominis* (Fig. 5B). Similarly, the ventral edge of the anterior lobe, which also contacts the water surface during a stroke, is lined with a band of 3–4 rows of *H1* setae (Fig. 4B9; Figure S16); however, only lateral views were available, limiting precise row counts. On ventral tarsomere 2, which also interacts with the water surface without breaking it (Figure S5), we observed three rows: a posterior row of *H1* setae and two anterior rows of *Sp* setae, separated by gaps (Fig. 4B12, B13; Figure S17).

In *G. latiabdominis*, a “gaps and rows” arrangement of setae was observed along ventral midleg sections that interact with the water surface (Fig. 5B; Figures S20A–F and S21A–C). This pattern resembles the structure on the ventral edge of the lobe in *R. distincta* (Fig. 4B9). It is especially prominent on the tarsus, where a main ventral gap separates a row of cuspidate setae (*C*) from a posterior row of thorn-like *T2* setae, with a second *T2* row positioned further posteriorly to form a *T2–T2* gap (Fig. 5B4–B9). Anterior to the *C* row, a row of *M1* setae creates a *C–M1* gap (Figure S20E). The tips of these three setae types bend distally along the leg`s longitudinal axis, particularly the cuspidate setae, whose long, flat distal sections may contact each other. In contrast, setae on the lateral and dorsal leg surfaces are relatively straight (Figure S21B). On tarsomere 2, a second, less regularly arranged anterior *M1* row creates an *M1–M1* gap (Figure S20C, E). This arrangement is less distinct on the tibia, where *T2* setae exhibit intermediate morphology between *T2* and *M1*, and the *C* setae are replaced by *M2* (Fig. 5B3). At the tip of the tarsus, the pattern disappears, replaced by a ventral concentration of grass-blade-like setae (*g*) (Fig. 5B12).

### Ventral microstructures for support and sliding

In *R. distincta*, specialized microstructures involved in support and sliding were found on the ventral tarsi of the forelegs (Fig. 4C), hindlegs (Fig. 4A), and at the distal tips of the midleg tarsus (Fig. 4B10, B11). These structures consist of one or more rows of *Sp* setae with flattened, bent tips that overlap to form a “beam-like” surface ~ 20–25 μm below the leg cuticle, oriented toward the water. Two *Sp* rows were observed on the ventral forelegs (Fig. 4C3, C4; Figure S18) and 1 on the hindlegs (Fig. 4A1; Figure S19), with the flattened tips especially prominent near the leg ends (Figs. 4A5, A6; 4C4). *Sp* rows are flanked—particularly posteriorly—by *H1* setae on the forelegs (Fig. 4C; Figure S18) and *M2* setae on the hindlegs (Fig. 4A; Figure S19). At the tips of the midlegs, which also provide support, a dense cluster of *Sp* setae—likely modified *H1* types—was observed, with long, flat, overlapping tips (Fig. 4B11; Figure S15F, G).

In *G. latiabdominis*, specialized microstructures involved in support and sliding were found on the ventral forelegs and hindlegs. On the ventral foreleg tarsus, a band of grass-blade setae (*g*) with overlapping flat, bent distal tips was observed ~ 15–20 μm from the leg cuticle, facing the water surface (Fig. 5C4; Figure S22). An entangled cluster of web setae (*W*) was also present, especially near the tibiotarsal joint (Fig. 5C2; Figure S22). On the ventral side of the hindleg tibia and tarsus, 2–3 rows of leaf-blade setae (*L*) were arranged in an orderly manner (Fig. 5A; Figure S23). Their overlapping distal tips formed a “beam-like” surface with nanogrooves, ~ 20 μm above the water surface (Fig. 5A2; Figure S23), and were accompanied by a posterior row of large thorn setae (*T3*) (Fig. 5A6, A8; Figure S23A, E). The ventral sides of joints were covered with bundles of *L* and leaf-like *l* setae (Fig. 5A1, A6), with *l* setae also present at the tarsal tips (Fig. 5A9).

### Contact angle on midleg sections used in thrust generation

**Fig. 6:**
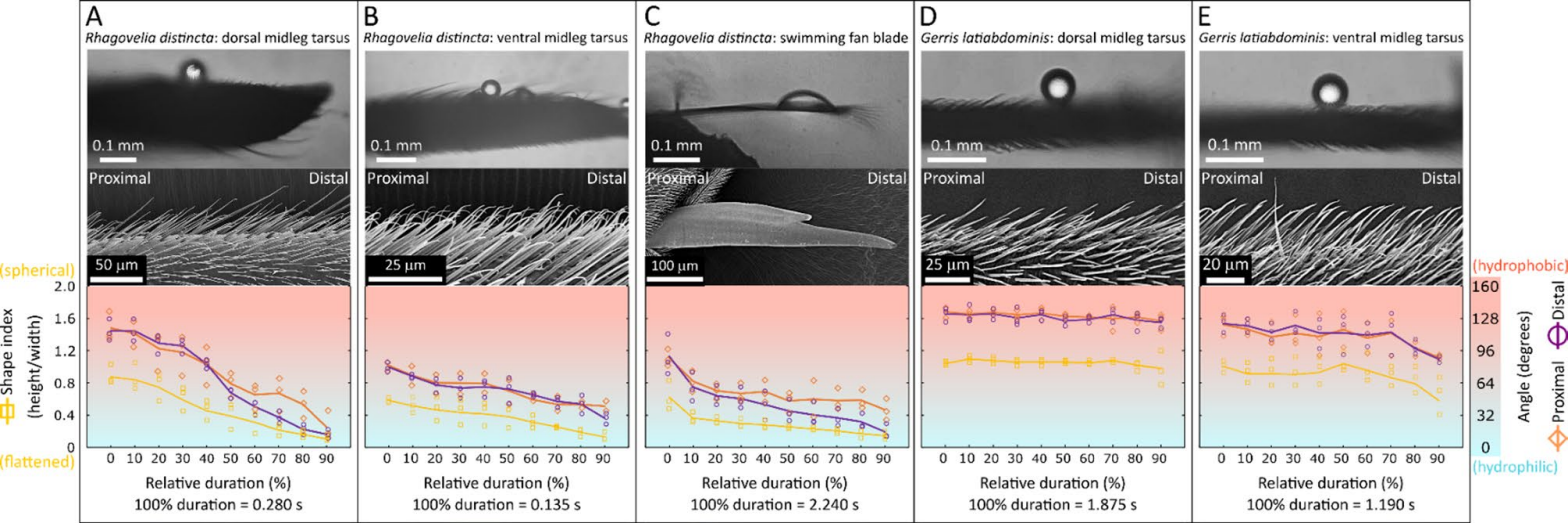
Contact angle and droplet shape on distal tarsus of *Rhagovelia distincta* and *Gerris latiabdominis*. Top panels are example images of water droplets on different leg surfaces. Middle panels contain corresponding SEM images showing dorsal hair layers (**A, D**), ventral hair layers (B, E), and the surface of *R. distincta*`s claw (**C**). Bottom panels are time-course changes (relative to droplet disappearance, set as 100%) in droplet shape index (height/width, yellow squares) and contact angle (degrees) measured on the proximal (orange diamonds) and distal (purple circles) sides of the droplet. (**A**) Contact angle on *R. distincta*`s dorsal midleg tarsus (tarsomere 3). (**B**) Contact angle on *R. distincta*`s ventral midleg tarsus (tarsomere 3). (**C**) Contact angle on *R. distincta*`s claw. (**D**) Contact angle on *G. latiabdominis*`s dorsal midleg tarsus (tarsomere 2). (**E**) Contact angle on *G. latiabdominis*`s ventral midleg tarsus (tarsomere). For additional details, see Figure S24 and commentary in SI Part 4, and Video S4.

Midleg tarsal surfaces in *G. latiabdominis* were generally more hydrophobic than those in *R. distincta* (Fig. 6). In *R. distincta*, water droplets rapidly lost their spherical shape after contacting the hair layer, spreading 10–40% and showing decreased shape indices. Contact angles on the dorsal and ventral midleg surfaces progressively decreased from approximately 130.3° and 75.5°, respectively, to as low as 21.9° as droplets collapsed, indicating relatively high surface wettability (Fig. 6A, B; Figure S24). This effect was especially pronounced on the ventral side, where the swimming fan and associated microstructures interact with water during locomotion (Fig. 6B, C; Figure S24). Contact angle measurements revealed that the ventral side of *R. distincta*`s midleg—particularly the region housing the swimming fan—exhibits clear hydrophilic properties, in contrast to the more hydrophobic surfaces observed in *G. latiabdominis*. This elevated wettability likely facilitates water surface penetration during fan deployment, reducing resistance and promoting stable submersion of the fan. Such localized wetting, combined with the fan`s structural stiffness, inferred from setae and setulae microstructure, may enhance the effectiveness of drag-based thrust generation. In contrast, droplets on the dorsal and ventral midleg surfaces of *G. latiabdominis* retained their spherical shapes with minimal spreading (shape index ≈ 1.5), and contact angles remained high throughout dissipation, ranging from 132.1° to 109.3°, consistent with strong hydrophobicity (Fig. 6D, E; Figure S24; SI Part 4). These uniformly hydrophobic surfaces align with *G. latiabdominis*` reliance on surface tension, supporting the leg`s ability to retain air layers and resist surface penetration during sliding or thrust.

### Kinematics profiles during a stroke

**Fig. 7:**
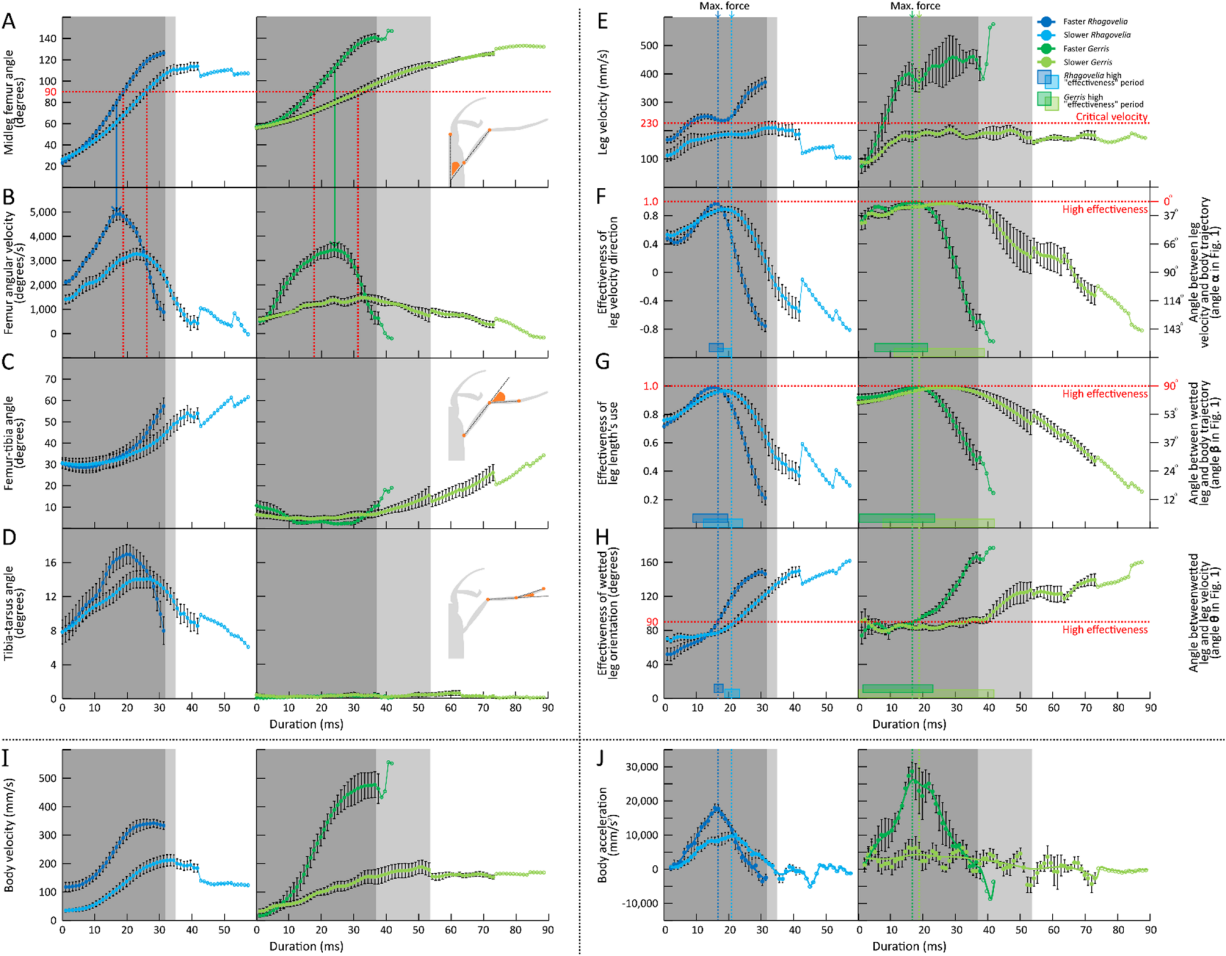
Kinematic profiles of ***Rhagovelia distincta*** and ***Gerris latiabdominis*** during a stroke. (**A**) Midleg femur angle (degrees ± SE): angle between femur and body axis; (**B**) Midleg angular velocity (degrees/s ± SE), derived from A. (**C**) Femur-tibia angle (degrees ± SE). (**D**) Tibia-tarsus angle (degrees ± SE). (**E**) Leg velocity (mm/s ± SE) along the direction of the wetted midleg trajectory on the water surface. (**F**) “Effectiveness” of leg velocity vector`s direction. (**G**) “Effectiveness” of leg length`s use. (**H**) “Effectiveness” of wetted leg orientation. (**I**) Body velocity (mm/s ± SE) along the body movement axis. (**J**) Body acceleration (mm/s^2^ ± SE), derived from I with spline curve fitted to the averages. Insets in (**A–D**) illustrate how each angle was measured: dark blue indicates fast strokes of *R. distincta*, light blue indicates slow strokes of *R. distincta*, dark green indicates fast strokes of *G. latiabdominis*, and light green indicates slow strokes of *G. latiabdominis*. Period of high “effectiveness” in (**F** and **H**) and (**G**) illustrate range of ± 0.1 and ± 10°, respectively, from each maximal “effectiveness” observed. Gray-shaded regions indicate time intervals where data from all individuals were included in the average; standard error is shown only when *n* > 4. Sample sizes: 21 strokes from six individuals of *R. distincta* and 12 strokes from six individuals of *G. latiabdominis*.

In both species (Fig. 7), the midleg femur angle increased gradually during the stroke, but more steeply in *R. distincta* than in *G. latiabdominis*. The initial angle was more acute in *R. distincta* (~ 20°) compared to *G. latiabdominis* (~ 60°), with both reaching a final angle of ~ 120° (Fig. 7A, B). Peak femur angular velocity was higher in *R. distincta* and occurred mid-stride at a femur angle of 85°, whereas in *G. latiabdominis* it peaked at 100° (Fig. 7A, B). In G. *latiabdominis*, the femur–tibia and tibia–tarsus angles remained relatively small throughout the stroke. In contrast, *R. distincta* showed more pronounced changes: the femur–tibia angle increased from 30° to 60° (Fig. 7C), and the tibia–tarsus angle ranged from 8° to 16° (Fig. 7D). These patterns suggest that *R. distincta* engages in coordinated rotations at the coxa–femur, femur–tibia, and—though to a lesser extent—tibia–tarsus joints, while in *G. latiabdominis*, motion is largely concentrated at the coxa–femur joint.

In both species, leg velocity during slower (longer) strokes remained below the theoretical critical velocity (~ 0.23 m/s; Fig. 7E), which marks a transition threshold above which thrust generating legs of semiaquatic organisms generate capillary-gravity waves to achieve higher propulsion force^11–13^. In faster (shorter) strokes, leg velocity exceeded this threshold within ~ 5 ms and reached higher peak values in *G. latiabdominis* than in *R. distincta* (Fig. 7E). In *R. distincta*, the leg velocity vector was briefly aligned with the body movement axis only during mid-stroke, when the femur was approximately perpendicular to the trajectory (Figs. 7G and 9G). For the remaining stroke, the fan–acting as an oar blade–moved in a direction misaligned with the body axis and not perpendicular to its movement direction. In contrast, in *G. latiabdominis*, the leg velocity vector remained nearly parallel to the body movement axis (Figs. 7F and 9E) and nearly perpendicular to the wetted leg section (Figs. 7H and 9G) for a much larger portion of the stroke (rectangles on x-axis). In both species, the highest net force per stroke, indicated by peak body acceleration (Fig. 7J), coincided with intervals of high “effectiveness” indices (Fig. 7F–H; SI Part 5).

### Kinematics comparisons of a stroke

**Fig. 8:**
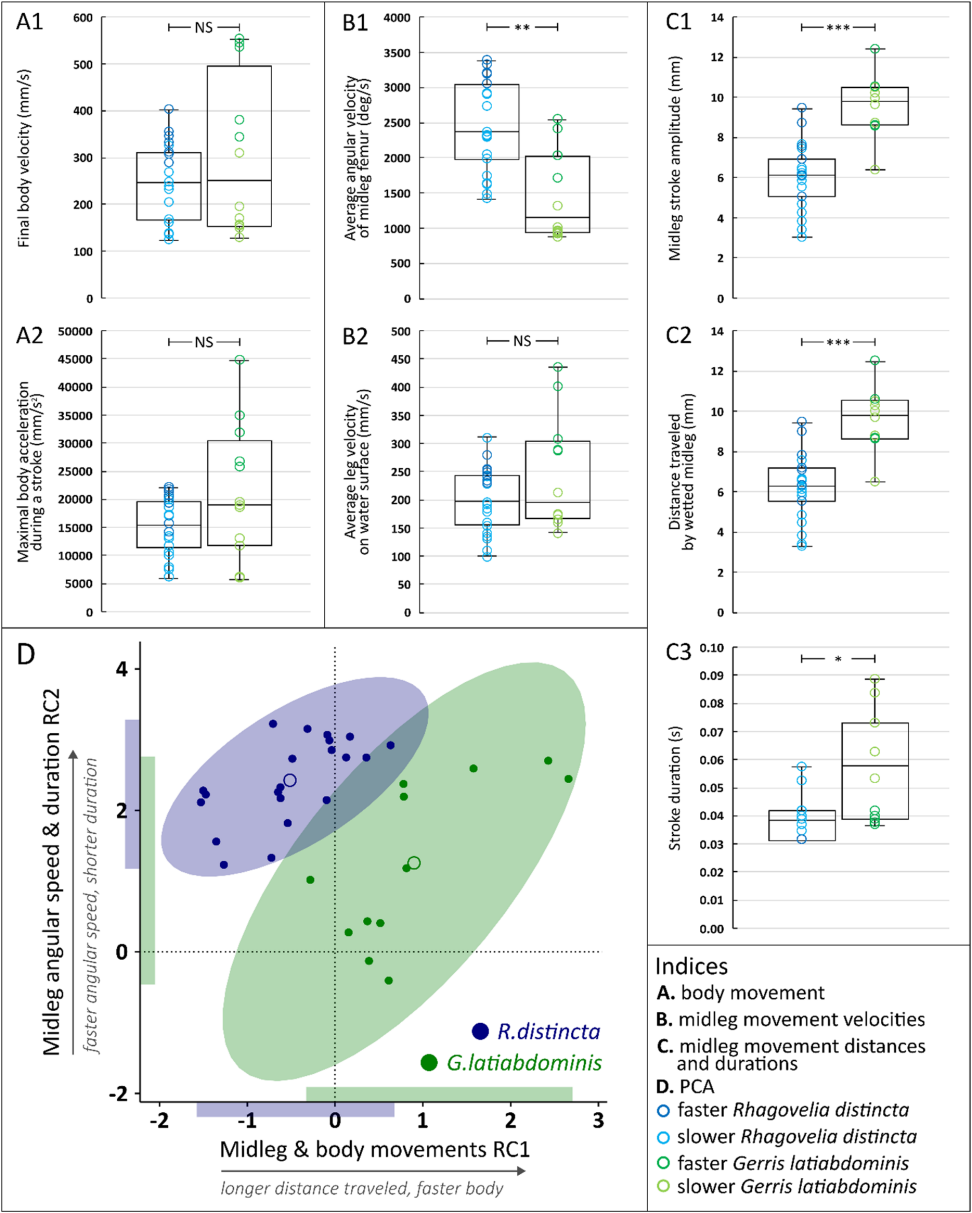
Comparative kinematics between *Rhagovelia distincta* and *Gerris latiabdominis* during a single thrust stroke. Each data point represents one of 21 (*R. distincta*) and 12 (*G. latiabdominis*) stroke observations from six individuals per species. (**A**) Body movement variables: (A1) final body velocity (mm/s); (A2) maximal body acceleration (mm/s^2^). (**B**) Midleg movement velocity variables: (B1) average femur angular velocity (degrees/s); (B2) average leg velocity on the water surface (mm/s). (**C**) Midleg movement distance and time variables: (C1) stroke amplitude of the midleg (mm); (C2) distance traveled by the wetted midleg (mm); (C3) stroke duration (s). (**D**) Principal Component Analysis (PCA): scatterplot of strokes based on two principal components (Table 1) extracted from variables in panels A–C. Ellipses represent 95% confidence intervals: *R. distincta* in blue, *G. latiabdominis* in green. RC1 corresponds primarily to distance and speed; RC2 to stroke duration and angular velocity. Shaded bands on x and y axes illustrate the ranges of observed values for each species.

A small subset of *G. latiabdominis* strokes showed notably higher average leg linear velocities, body velocities, and peak accelerations (Fig. 8). However, when all strokes were analyzed together, there were no significant differences between the two species in average midleg velocity (Fig. 8B2; Table S4), final body velocity (Fig. 8A1; Table S4), or maximal body acceleration (Fig. 8A2; Table S4). Strokes by *R. distincta* were shorter in duration (Fig. 8C3; Table S4) and involved faster angular femur movements (Fig. 8B1; Table S4). The legs traveled a shorter distance across the water surface during each stroke compared to *G. latiabdominis* (Fig. 8C1, C2). This difference is notable because *R. distincta* has shorter legs (Tables S1 and S2), and additional angular movements were observed at the tibia and tarsus (Fig. 7C, D). Since the kinematic variables are intercorrelated (Figure S26D), estimates of their individual effects on body speed (Figure S26A–C; Tables S5, S6 and S7) are not independent. To address this, we performed a principal component analysis and extracted two components (Table 1). Strokes by *R. distincta* were characterized by shorter duration and higher femur angular velocity (higher RC2 values), along with shorter distance and slower leg speed on the water surface (lower RC1 values) (Fig. 8E), distinguishing them from *G. latiabdominis*.

### Estimated force output for thrust generation

Although we could not directly measure resistance forces during sliding and thus cannot determine total thrust, the body acceleration profiles during stokes allowed us to estimate the net horizontal thrust vector and the peak net force generated per stroke (Figure S25). Given that approximately 85–95% of total thrust typically translates into forward body momentum in water striders^12^, our net force estimates likely underestimate the true thrust force by 5–15%.

In *R. distincta*, the maximum horizontal net force per stroke averaged 152 µN with an absolute maximum of 324 µN (Figure S25C). This force was generated through the symmetrical action of two pretarsal swimming fans, each with a projected area of 0.89 ± 0.04 mm^2^ (1.78 mm² total; Figure S3), along with contribution from wetted tarsi measuring 1.80 ± 0.06 mm (Table S1) in length. Assuming the primary thrust originates from the fans, this translates to an average force of 85 µN/mm² of fan surface area per stroke with an absolute maximum of 183 µN/mm².

In *G. latiabdominis*, the maximum horizontal net force per stroke average 360 µN with an absolute maximum of 745 µN (Figure S25D). This force was generated using wetted midlegs that depress the water surface to create dimples, without piercing it. Based on average wetted leg length of 7.76 ± 0.11 mm (Table S2), this corresponds to an average of 24 µN/mm of leg length with an absolute maximum of 48 µN/mm. To compare per-unit-area force outputs, we approximated the wetted leg segment as a cylindrical surface. With a midleg diameter ranging from 80 to 110 μm (Figures S20 and S21), we used an average radius of 0.05 mm. Assuming half the lateral surface of a 1 mm-long cylinder interacts with the water, the effective thrust-generating area is ~ 0.157 mm^2^. Based on this, *G. latiabdominis* generated an average of 148 µN/mm² of interacting leg surface with an absolute maximum of 305 µN/mm².

These estimates reflect functional differences in thrust-generation strategies: drag-based propulsion in *R. distincta* through fan employment and surface-tension-based propulsion in *G. latiabdominis* through longitudinal row-and-gaps ventral setal structures engagement. Importantly, these values offer a quantitative baseline for future mechanical or computational modeling efforts aimed at linking leg microstructure to propulsion performance.

**Table 1:** Principal component analysis of behavioral variables. PCA was conducted on seven behavioral variables from *R. distincta* (*n* = 21) and *G. latiabdominis* (*n* = 12). The table shows eigenvalues, percentage of variance explained, and loadings for the first two rotated components (RC1 and RC2), based on the *fa.parallel* and *principal* functions from the **psych** R package. Loadings with absolute values greater than 0.75 are shown in bold. Related results are illustrated in Fig. 8D.

	RC1 Midleg`s & Body`s Movements PC	RC2 Midleg Angular Speed & Duration PC
Linear velocities & accelerations		
Average midleg velocity (mm/s)	**0.80**	0.53
Maximum acceleration (mm/s^2^)	**0.77**	0.48
Final body velocity (mm/s)	**0.77**	0.60
Linear midleg movement distances		
Midleg`s stroke amplitude (mm)	**0.96**	−0.22
Distance traveled by wetted midleg (mm)	**0.96**	−0.20
Angular midleg (femur) speed and duration		
Average angular velocity (degrees/s)	0.03	**0.93**
Stroke duration (s)	0.04	**−0.98**
Eigenvalue	3.67	2.77
Variance %	52%	40%

## Discussion

Observations of live *R. distincta* suggest that thrust during a stroke results from a combination of two forces: hydrodynamic forces generated by the oar-like motion of the fan, as proposed in previous studies^5,7,9^, and additional capillary forces arising from an anteroposteriorly asymmetrical dimple beneath the tarsus, consistent with surface-tension-based mechanisms described in Gerridae^6^. In contrast, behavioral evidence from *G. latiabdominis* aligns with the surface-tension-based thrust mechanism characterized in detail for *Aquarius paludum*^6^. The observed differences between these species appear closely tied to variations in leg microstructures, respective internal nano-structural properties, and motion kinematics that reflect their distinct ecological contexts and thrust-generation strategies.

In *R. distincta*, several microstructural features suggest specialization for hydrodynamic thrust. The hydrophilic properties of the claw likely facilitate surface penetration during stroke initiation, as the fan and claw extend downward from the cleft`s internal compartment. Similar surface textures between the claw and fan setae indicate that the fan may also be hydrophilic, enhancing its ability to submerge through the water surface. The fan`s setae and setulae are oriented to press against the water with their narrow edges, a configuration that minimizes deformation under hydrodynamic forces, as predicted by beam theory^14^ (SI Part D).

The internal architecture of the fan setae and setulae appears well-adapted for their role as underwater oars in thrust generation. Each seta comprises a hollow core reinforced with columnar nanofibers, while the associated claw features a lamellar structure—both resembling engineered designs such as sandwich and lamellar composites^15,16^, which are known to enhance stiffness and fatigue resistance. Polymer nanofibers are widely recognized for their high strength due to a high surface-area-to-volume ratio, and thinner fibers are particularly associated with greater flexibility and mechanical resilience^17,18^. In *R. distincta*, the small diameter of nanofibers within the internal walls of these flat and hollow beams likely contributes to their flexibility. Combined with the hydrophilic properties of the surface, this structure may underlie the elastocapillary behavior responsible for the fan`s characteristic “fan-like” shape when immersed in water—an effect observed both in our experiments (SI Part 3B; Figures S8 and S9) and in Ortega-Jimenez et al. (2025). Furthermore, the multidirectional growth pattern of nanofibers likely reinforces the setae across multiple planes, enhancing their ability to resist water forces. As with synthetic hollow nanofiber systems^19^, the central hollow core may facilitate stress distribution, controlled deformation under load, and reduced material weight—all contributing to the fan`s structural integrity and functional performance resulting in net thrust outputs of ~ 300 µN per stroke and 180 µN/mm^2^ of fan surface area.

Additionally, the H1 and H2 setae rows positioned at the cleft entrance likely act as a barrier against water intrusion while assisting fan deployment through elastocapillary interactions. Based on the observed stroke speed, setula spacing, and thickness, we estimate the fans to operate at Reynolds numbers (dimensionless quantity expressing the ratio of inertial to viscous forces in a fluid) ranging from ~ 0.03 to 0.20 (Table S8) and fan`s leakiness (degree to which fluid flows through, rather than around, a porous or bristled structure, depending on geometry and flow regime) to range from ~ 0.3 to 0.6 (Figure S27), suggesting it behaves as a “leaky paddle” rather than a solid blade. This is roughly like bristled appendages in copepods and barnacle larvae^20,21^. Comparable fan-like structures with potential “leaky paddle” functionality are found in other Veliidae genera such as *Tetraripis* and *Trochopus*. In contrast, “Veliidae” species that depend primarily on surface-tension-based thrust, like *Velia sp.*, tend to exhibit more developed ventral “gaps and rows” arrangements and less-developed fan structures^5^, supporting the hypothesis of divergent functional adaptations.

In *G. latiabdominis*, a different set of microstructural adaptations supports thrust generation primarily through surface tension. The species exhibits denser and more hydrophobic setae on its midlegs than *R. distincta*, likely reflecting its reliance on surface-tension-based propulsion. Deep, asymmetrical dimples beneath the wetted portion of the leg, along with prominent bow waves, contribute to increased thrust. Interestingly, even the lower hydrophobicity observed in *R. distincta* can still support thrust via surface tension through dimple formation, though to a lesser degree.

Across both species, leg surfaces involved in surface-tension-based thrust display linear arrangements of distally bending setae forming “gaps and rows.” These are absent from other leg surfaces and may serve specialized functions, potentially related to air retention during dimple formation, which prevents surface penetration and facilitates thrust. Theoretical work^22,23^ suggests such arrangements can trap air and maintain smooth water contact, providing theoretical support for our hypothesis that these structures appear to function like pressurized air pockets that resist water surface breakage under thrust loads. Additionally, the smoother longitudinal gaps may reduce adhesion during stroke recovery. This arrangement is more pronounced in *G. latiabdominis*, where the setae also exhibit nanogrooves known to enhance hydrophobicity^24,25^. The posterior concentration of thicker T2 and T3 setae in mid- and hindlegs may be a specific adaptation to withstand the higher pressure during backward strokes, which is exemplified by higher thrust output per leg surface area in *G. latiabdominis* than *R. distincta*. These structural reinforcements are aligned with previous findings on jumping and propulsion in surface-dwelling insects^26,27^.

Kinematic data further underscores how thrust generation mechanisms are integrated with species-specific leg movement strategies. In *R. distincta*, hydrodynamic thrust is supported by a midleg stroke that begins from a more acute femur angle and involves backward rotations across multiple leg joints. The resulting fan movement vectors deviate from the body movement axis similar to patterns characteristic to *Xenopus* frogs, known to incorporate lift^28^. This suggests potential contribution of lift-like forces to thrust, akin to paddling strategies in human kayaking^29,30^ and animal locomotion^28,31^. The observed combination of *Rhagovelia*`s leg movement pattern and strokes of shorter duration would be inefficient for surface-tension-based propulsion on stagnant waters but is well-suited to fast-flowing environments where high-frequency of short strokes with minimal surface contact time is advantageous. The ability of *R. distincta* to actively control fan protraction and retraction through muscle action^5^ (SI Parts 2 and 3B) could allow for greater flexibility during maneuvering and stroke timing, compared to the hypothetical passive fan deployment proposed in recent studies of another *Rhagovelia* species^8,9^, but not supported by our observations.

Conversely, *G. latiabdominis* initiates midleg movement at a less acute femur angle, with primary rotation occurring at the coxa–femur joint, and maintains a nearly straight femur–tibia segment. This configuration enhances wetted surface area, allowing for more efficient surface-tension-based thrust. The leg stroke is longer in duration and follows a nearly parallel trajectory to the body axis, facilitating the formation of asymmetrical dimples critical to curvature force production. The backward movement of the leg, nearly perpendicular to its axis, promotes directional asymmetry in the dimple, optimizing forward propulsion typical for this species.

Forelegs and hindlegs support the insect body during thrust and sliding, and both species share a distinct ventral setal arrangement that likely contributes to standing and sliding on the water surface. Flattened, overlapping setae form a beam-like structure along the underside of wetted legs, similar to those described in *Gerris* and *Aquarius*^5,10^. This configuration minimizes surface penetration and drag, offering support without water breakage. The beam`s alignment with body motion also likely facilitates sliding and steering. In the heavier *G. latiabdominis*, these beams feature hydrophobic nanogrooves that enhance their supporting function. In contrast, the lighter *R. distincta* lacks such grooves, suggesting lower support demands. Moreover, the spoon-like setae at the tips of *R. distincta*`s midlegs may help resist displacement by currents, offering a stabilizing advantage in fast-flowing habitats.

## Conclusions

Our results demonstrate that *Rhagovelia distincta* and *Gerris latiabdominis*, despite both having independently evolved symmetrical rowing, employ fundamentally different thrust-generation strategies: *R. distincta* relies primarily on hydrodynamic drag (and possibly lift) via actively controlled pretarsal fans functioning as “leaky paddles,” while *G. latiabdominis* generates thrust through surface tension. Detailed morphological and kinematic analyses revealed that fan protraction and retraction in *R. distincta* involve muscle action, whereas elastocapillarity contributes only to fan conformation once submerged^9^. The fan`s nanostructured architecture supports this function through its stiffness, flexibility, and surface wettability. We also show that both species exhibit morphological differences linked to their respective thrust strategies and similarities in ventral microstructures—such as longitudinal setal rows-and-gaps and beam-like setal structures—linked to the mechanisms of support and sliding on water surface. These findings challenge simplified models of leg micromorphology in surface-dwelling insects and suggest that precise microstructural arrangements are critical to locomotor performance. By providing detailed, testable hypotheses on structure-function relationships, our study lays a foundation for future mechanical modeling and for comparative evolutionary analyses connecting microstructure, movement, and ecological adaptation in semiaquatic bugs.

## Methods

### Field sites and study species

**Fig. 9:**
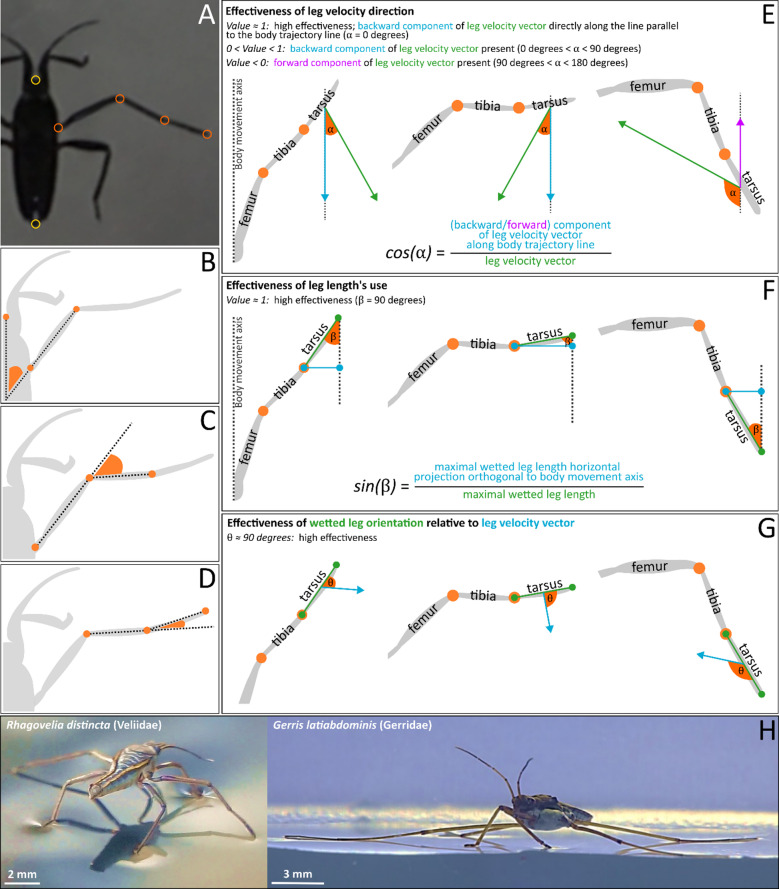
Graphical definitions of variables extracted from video recordings of thrust-generating strokes. (**A**) Digitized tracking points on the insect body and midleg. (**B–D**) Joint angles: (**B**) femur angle relative to the body axis, (**C**) femur-tibia angle, and (**D**) tibia-tarsus angle. (**E–G**) Indices of “effectiveness” for midleg orientation and movement: (**E**) effectiveness of leg velocity direction, (**F**) effectiveness of leg length`s use, (**G**) effectiveness of wetted leg orientation. Further details are provided in the ‘Methods’ section. (**H**) Side-by-side images of study species–*Rhagovelia distincta* and *Gerris latiabdominis*.

In January and February of 2020 and 2023, specimen collections and detailed observations of *Rhagovelia distincta* (body weight: 4–14 mg; Fig. 9H; Table S1) were made at the Southwestern Research Station, Arizona, USA (SWRS; 31°53′3′′N, 109°12′21′′W). In August and September of 2020, specimen collections and detailed observations of *Gerris latiabdominis* (16–19 mg; Fig. 9H; Table S2) were made at Gwanak Mountain, Korea (37°26′42′′N, 126°57′51′′E) and Seoul National University, Korea (37°28′57′′N, 126°96′04′′E), respectively. Each individual was weighed (GEM20 High Precision Digital Milligram Jewelry Scale, Smart Weigh, 0.001 g).

### Videographic and photographic observations

We filmed four types of high speed and standard videos with Sony RX10-III at 959.04 frames per second (fps) and with Chronos 2.1-HD at 1000–4000 fps of individuals in acrylic containers (18 × 18 cm, filled with water). These standardized still-water conditions were maintained to enable direct, quantitative comparison of kinematic and microstructural variables between species. The strength of this approach is that it removed environmental variability and allowed the isolation of species-specific locomotor traits shaped by their respective ecological conditions.

- **Type 1**: directly from above (85 and 62 movies collected from six and seven individuals of *R. distincta* and *G. latiabdominis*, respectively).
- **Type 2**: from the side of various angles (below surface, surface level, and above surface) (249 and 87 movies of *R. distincta* and *G. latiabdominis*, respectively).
- **Type 3**: directly from below with light source positioned directly above the container (20 and 60 movies from two and five individuals of *R. distincta* and *G. latiabdominis*, respectively) to visualize the shadows on the bottom of the container; shadows correspond to dimples under legs on the water surface.

Two variables were extracted from the video types 2 and 3:

- ***Wetted midleg length*** **(mm)**: maximal leg section in contact with the water surface in the middle of fast thrust strokes when midleg angle to body axis approximates 90°. The wetted midleg consisted of tarsus in *R. distincta* (6 individuals) and proximal tibia to tarsal tip in *G. latiabdominis* (6 individuals).
- ***Projected swimming fan area*** **(mm**^**2**^**)**: fan surface area was estimated from six still frames taken from six type 2 clips of *R. distincta*, where the midleg tarsus was perpendicular to both the body axis and camera lens axis (Figure S3). Projected area was calculated assuming the fan forms a circular sector with radius equal to the average of six measured setal lengths. As the fan surface is tilted ~ 80° to the lens (rather than 90°), projections slightly underestimate true area. However, since tilt angles for individual frames were unavailable and the bias is minor, we used the projected area as our estimate.

### Microscopic observations of leg microstructures

Using optical microscopy, we observed the morphology and behavior of the fan in specimens of *R. distincta*. Using Scanning Electron Microscopy (SEM), we visualized the hair structures on leg sections that interact with water (SI Part 3).

### Contact angle measurements (SI part 4)

Contact angle (in degrees; °), height and width (mm), and respective shape index (height to width ratio) of small droplets (0.096 ± 0.032 mm) on the surface of ventral and dorsal microstructures of tarsomere 3 and tarsomere 2 of *R. distincta* and *G. latiabdominis*, respectively, and on the tarsal claw of *R. distincta*, were measured (with ImageJ 1.53t) in frames of three high-speed videos per condition (2000 fps; Chronos 2.1-HD Camera, Kron Technologies). The specimens, sprayed with water, were mounted on a micromanipulator (MM-3, Narishige, Japan) parallel to the camera (Video S4).

### Kinematic profiles of a stroke

Detailed kinematic analyses of symmetric strokes by *R. distincta* and *G. latiabdominis* were restricted to data extracted with *Tracker* (https://physlets.org/tracker/) from selected videos: 21 and 12 strokes from six and six individuals for *R. distincta* and *G. latiabdominis*, respectively (Supplemental data 1; Supplemental data 2). All strokes were chosen based on strict inclusion criteria—specifically, individuals had to remain stationary before initiating a straight, uninterrupted thrust—to ensure consistent, high-quality data suitable for accurate 2D digitization and meaningful interspecific comparisons. Cartesian (*x*, *y*) coordinates of 10 and 9 points on the insect body for *R. distincta* and *G. latiabdominis*, respectively (midleg tips were not digitized in *G. latiabdominis* due to resolution issues) were digitized and subsequently smooth-splined using the “*stats*” package^32,33^ (df = 5 and smoothing parameter = 0.5).

To compare the two species, we focused on five aspects (Fig. 9) of leg movements during a stroke and extracted the following kinematic variables for each frame, or two consecutive frames, through the thrust phase of a stroke:

- ***Midleg femur angl*e (degrees)**: the angle between the body axis and the femur at the coxae (Fig. 9B) was calculated at each frame. The coxal joint is where the major leg angular movement is performed in both species.
- ***Femur-tibia angle*** **(degrees)**: the angle between the femur and the tibia at the femorotibial joint was calculated at each frame (Fig. 9C).
- ***Tibia-tarsus angle*** **(degrees)**: the angle between the tibia and the tarsus at the tibiotarsal joint was calculated at each frame (Fig. 9D).
- ***Midleg angular velocity*** **(degrees/s)**: calculated by dividing the between-frame difference in midleg femur angles by the latency between the two consecutive frames (i.e., 1/fps).
- ***Leg velocity*** **(mm/s) (*U*)**: the linear velocity in horizontal plane of the midpoint of the wetted midleg length. It is calculated for each pair of two consecutive frames via dividing the distance traveled between by the latency by the two consecutive frames.

Three proxies of “effectiveness” of midleg application for thrust generation during a stroke were calculated (Fig. 9) using basic trigonometry and vector algebra:

- ***“Effectiveness” of leg velocity vector`s direction*** (Fig. 9E) (proportion; range 0–1): the proportion of the leg velocity vector (and of *R. distincta*`s fan protracted under the leg; green vector) employed along the direction parallel to the body movement (blue or violet vectors). Values closer to ‘1’ indicate “more effective” employment of legs on water surface because the backward leg velocity vector is near-parallel to the body axis line ($\:{\upalpha\:}=0^\circ\:$) resulting in anteroposterior asymmetry of the dimple crucial for curvature force (i.e., surface tension) contribution to thrust^6^. Positive values indicate backward velocity vector (blue) that contributes to forward thrust, while negative values indicate forward vector (violet; when legs are dragged along body movement).
- ***“Effectiveness” of leg length`s use*** (Fig. 9F) (proportion; range 0–1): evaluation of the relative length of wetted leg projection (blue) on the line perpendicular to the body movement axis (relative to the actual wetted leg length marked green). In *G. latiabdominis*, it may be approximately viewed as the effective proportion of the total wetted midleg length (blue) that pushes the surface dimple directly backwards along the leg velocity vector parallel to the body movement direction (blue vector in Fig. 9E). Values closer to ‘1’ indicate “more effective” employment of the midleg length pushing the dimple backwards; they also indicate that the fan surface in *R. distincta* is approximately perpendicular to the body axis ($\:{\upbeta\:}\approx\:90^\circ\:$), under the assumption that tarsus on the water surface lies approximately within the near-vertical plane with the surface of the *R. distincta`s* fan under water.
- ***“Effectiveness” of wetted leg orientation*** (Fig. 9G) (degrees): angle θ indicates the orientation of wetted midleg`s main axis (as well as the plane of the fan protracted under the leg, assuming fan surface`s plane includes the longitudinal axis of wetted leg) relative to the leg velocity vector. Angles closer to ‘90°’ indicate “more effective” employment of the full wetted midleg length in pushing the water surface dimple along the leg velocity vector and creating dimple asymmetry along the velocity vector. Under the assumption that longitudinal axis of wetted leg on the water surface approximately lies within the plane of the *R. distincta* fan`s surface protracted under water, θ values closer to 90° indicate that the angle between the fan surface and the fan movement direction is near perpendicular and hydrodynamic drag from the fan pushing backwards contributes to thrust.

We additionally extracted three variables from the body movements :

- ***Body velocity*** **(mm/s)**: distance (mm) traveled by the body center (position derived from the average of head and abdomen tip positions) between consecutive frames divided by the latency between the two consecutive frames (1/fps).
- ***Body acceleration*** **(mm/s**^**2**^**)**: rate of change of body velocity derived from each pair of consecutive body velocity values divided by the latency between the two frames.
- ***Net force*** **(µN)**: body acceleration (mm/s^2^) multiplied by insect body mass (mg) and by 0.001 for unit conversion. It represents a horizontal vector of net thrust force during a stroke.

### Kinematic characterization of a stroke

The following kinematic variables were extracted from 21 strokes of six individuals of *R. distincta* and 12 strokes of six individuals of *G. latiabdominis* (1 value per stroke):

- ***Distance traveled by wetted midleg (mm)***: sum of frame-by-frame distances of wetted midleg midpoint was measured along the actual trajectory of the midpoint during the thrust phase of a stroke.
- ***Midleg`s stroke amplitude (mm)***: direct straight-line distance from the initial (beginning of thrust stroke) to the final (end of thrust stroke; when midleg`s velocity vector is no longer opposite to the body velocity vector) positions of wetted midleg midpoint was measured.
- ***Average angular velocity (degrees/s)***: mean of all frame-by-frame midleg angular velocities calculated within the thrust phase of a stroke.
- ***Average midleg velocity (mm/s)***: mean of all frame-by-frame leg velocities calculated within the thrust phase of a stroke.
- ***Leg velocity at maximum body acceleration (mm/s)***: leg velocity that corresponds to the maximum body acceleration (i.e., maximum horizontal net force) within the thrust phase of a stroke.
- ***Maximal body acceleration (mm/s***^***2***^***)***: maximum value of body acceleration within the thrust phase of a stroke.
- ***Maximal net thrust force (µN)***: maximum value of body force within the thrust phase of a stroke.
- ***Final body velocity (mm/s)***: final value of body velocity (between the last two consecutive frames) in the thrust phase of a stroke.
- ***Midleg thrust duration (ms)***: latency from the initiation of midleg thrust movements to the moment of their disengagement.

### Statistical analyses

All analyses were performed in R version 4.3.1^33^. We used linear mixed-effects models (with ***“individual”*** as the random factor; “*lme4*” package^34^; “*lmerTest*” package^35^ to compare the effects of ***average leg velocity***, ***stroke duration***, and ***distance traveled by wetted midleg*** on the ***final body velocity*** between the two species (interaction with categorical variable “***species***”). However, these independent variables were correlated, and the statistical models would not allow proper evaluations of these effects. Therefore, we extracted principal components using functions *fa.parallel* and *principal* from the “*psych*” R package^36^ from the pooled data for both species considering all seven kinematic variables: ***body velocity***, ***maximum acceleration***, ***average angular leg velocity***, ***average leg velocity***, ***midleg`s stroke amplitude***, ***distance traveled by wetted midleg***, and ***stroke duration***. We focused on the variables with loading values > 0.75^37^.

## Supplementary Information

Below is the link to the electronic supplementary material.

Supplementary Material 1

Supplementary Material 2

Supplementary Material 3

Supplementary Material 4

Supplementary Material 5

Supplementary Material 6

Supplementary Material 7

## Data Availability

All data generated or analyzed during this study are available in supplementary files.
