# Supplementary material for: Distinct kinematics and micromorphology for symmetrical rowing and sliding on water in ripple bugs and water striders
Source: Sci Rep. 2025 Dec 29;15:44777. doi: 10.1038/s41598-025-28453-0 (PMC12749290; doi:10.1038/s41598-025-28453-0)
Supplement: Supplementary file 3 — Supplementary Material 3 [file 41598_2025_28453_MOESM3_ESM.docx]

SUPPORTING INFORMATION TO:

**Distinct kinematics and micromorphology for symmetrical rowing and sliding on water in ripple bugs and water striders**

Sang Yun Bang^1^, Woojoo Kim^1,2,3^, Jeongseop Lee^1^, Jinseok Park^1,3^, Varsha Khare^4^, Sang-im Lee^c5^, Piotr Grzegorz Jablonski^c1,6^

PART 1. Macro-morphology of the studied species

Tables S1, S2, Fig. S1

PART 2. Behavioral observations of live insects

Figures S2, S3, S4, S5

Commentary to Figures S3, S4, S5

PART 3. Microscopy and micro-morphology

3A) Optical microscopy and SEM – details of methods

3B) Microscopic dissection and experiments with dissected fan: Fig. S6, S7, S8, S9

3C) Types of setae – details: Table S3, Fig. S10, Commentary to Fig. S10 & Table S3

3D) SEM of Rhagovelia midlegs: Fig. S11, S12, S13, S14, S15, S16, S17

3E) SEM of Rhagovelia forelegs and hindlegs: Fig. S18, S19

3F) SEM of Gerris midlegs: Fig. S20, S21

3G) SEM of Gerris forelegs and hindlegs: Fig. S22, S23

PART 4. Contact angles

Fig. S24 , Commentary to Fig. S24

PART 5. Evaluation of horizontal net forces: Fig. S25

PART 6. Kinematics – statistical results

6A) Comparison of strokes: Table S4,

6B) Effect of leg kinematic variables on body velocity

Fig. S26, Tables S5, S6, S7

PART 7. Evaluation of Reynolds number for *Rhagovelia*’s fan

7A) Evaluation of *Re*: Table. S8

7B) Evaluation of leakiness: Fig. S27

PART 8. Descriptions of Supplementary Videos

VideoS1, VideoS2, VideoS3, Video S4

PART 9. References

`

**PART 1. Macro-morphology of the studies species**

*Rhagovelia distincta (Veliidae)*

*Gerris latiabdominis (Gerridae)*

tibia

tarsus

femur

femur

tibia

tarsus

tibia

tarsus

femur

tibia

tarsus

femur

wetted foreleg

tibia

wetted hindleg

*t1*

*t2*

*t3*

wetted midleg

wetted hindleg

wetted foreleg

*t2	t1*

tarsus

wetted midleg tip

*t1	t2*

**A**

**B**

2 mm

2 mm

**Figure S1. Study subjects on water surface.** The letters *t1, t2, t3* indicate tarsomere 1, 2 and 3 , respectively. “Wetted leg” refers to the leg sections that remain in touch with the water surface.

**Table S1. Morphological data of *Rhagovelia distincta*.** Morphological measurements of six individuals of *Rhagovelia distincta* extracted from Videographic material Type 1: body length (mm), leg segment lengths (femur, tibia, tarsus) (mm), maximal wetted leg length (Max. WL, mm; “wetted length” is the length of leg section in touch with water surface), and body mass (mg). For each individual, 2-7 clips were used to estimate the morphology of each individual. The means from all measurements on the 6 individuals are presented in the bottom rows (bold; mean ± SD). The foreleg femur measurements were excluded from the analysis as they were obscured by the insect’s head and antennae in the videos. Body mass was directly measured on the scale. Measurements for Individual 1 were taken from video clips C0001, C0010, C0012, C0013, C0015, C0017, C0018; for Individual 2 from clips C0025, C0026, C0027, C0028, C0032; for Individual 3 from clips C0036, C0041, C0045; for Individual 4 from clips C0079, C0085; for Individual 5 from clips C0114, C0119; for Individual 6 from clips C0124, C0125. Video clip ID indicates the name of specific video clip with the analyzed strokes in our archives.

| **IND ID**  *(nr of clips)* | **Body length (mm)** |  | **Femur length**  **(mm)** | | **Tibia length**  **(mm)** | | **Tarsus length**  **(mm)** | | **Max. WL of one leg**  **(mm)** | **Mass**  **(mg)** |
| --- | --- | --- | --- | --- | --- | --- | --- | --- | --- | --- |
|  |  |  | Left | Right | Left | Right | Left | Right |  |  |
| 1.  *(n=7 clips)* | 5.75±0.08 | Foreleg | - | - | 1.3±0.08 | 1.34±0.08 | 0.48±0.03 | 0.48±0.04 | 0.48±0.04 | 14.0 |
|  |  | Midleg | 2.2±0.08 | 2.2±0.10 | 1.9±0.06 | 1.90±0.05 | 1.84±0.02 | 1.84±0.04 | 1.84±0.03 |  |
|  |  | Hindleg | 1.8±0.04 | 1.7±0.07 | 2.1±0.04 | 2.01±0.07 | 0.82±0.03 | 0.84±0.03 | 0.83±0.03 |  |
| 2.  *(n=5 clips)* | 5.43±0.03 | Foreleg | - | - | 1.3±0.08 | 1.31±0.05 | 0.44±0.05 | 0.47±0.03 | 0.46±0.04 | 9.0 |
|  |  | Midleg | 2.4±0.12 | 2.4±0.05 | 1.9±0.08 | 2.01±0.13 | 1.80±0.05 | 1.74±0.07 | 1.77±0.07 |  |
|  |  | Hindleg | 1.7±0.09 | 1.7±0.08 | 1.9±1.41 | 1.90±0.13 | 0.82±0.05 | 0.79±0.05 | 0.81±0.05 |  |
| 3.  *(n=3 clips)* | 5.54±0.08 | Foreleg | - | - | 1.2±0.03 | 1.13±0.05 | 0.43±0.02 | 0.48±0.05 | 0.46±0.04 | 15.0 |
|  |  | Midleg | 2.3±0.13 | 2.3±0.10 | 1.8±0.04 | 1.81±0.07 | 1.70±0.04 | 1.72±0.04 | 1.71±0.04 |  |
|  |  | Hindleg | 1.8±0.05 | 1.8±0.05 | 2.0±0.11 | 2.00±0.03 | 0.78±0.05 | 0.77±0.05 | 0.78±0.04 |  |
| 4.  *(n=2 clips)* | 4.67±0.05 | Foreleg | - | - | 1.1±0.00 | 1.11±0.01 | 0.45±0.05 | 0.43±0.05 | 0.44±0.04 | 4.0 |
|  |  | Midleg | 2.5±0.08 | 2.5±0.23 | 1.9±0.03 | 1.88±0.14 | 1.85±0.01 | 1.85±0.04 | 1.85±0.02 |  |
|  |  | Hindleg | 1.7±0.05 | 1.8±0.01 | 2.0 ± 0.01 | 2.00 ± 0.04 | 0.75 ± 0.05 | 0.80 ± 0.07 | 0.77 ± 0.06 |  |
| 5.  *(n=2 clips)* | 4.84±0.02 | Foreleg | - | - | 1.1 ± 0.06 | 1.04 ± 0.06 | 0.44 ± 0.05 | 0.43 ± 0.03 | 0.44 ± 0.03 | 5.6 |
|  |  | Midleg | 2.5±0.10 | 2.6±0.01 | 2.0 ± 0.04 | 1.96 ± 0.04 | 1.83 ± 0.01 | 1.79 ± 0.01 | 1.81 ± 0.02 |  |
|  |  | Hindleg | 1.8±0.05 | 1.9±0.03 | 2.0 ± 0.06 | 1.94 ± 0.03 | 0.71 ± 0.02 | 0.72 ± 0.08 | 0.71 ± 0.05 |  |
| 6.  *(n=2 clips)* | 4.85±0.09 | Foreleg | - | - | 1.0 ± 0.03 | 1.07 ± 0.01 | 0.42 ± 0.02 | 0.46 ± 0.02 | 0.44 ± 0.03 | 4.6 |
|  |  | Midleg | 2.4±0.01 | 2.5±0.04 | 2.04 ± 0.04 | 1.99 ± 0.08 | 1.78 ± 0.00 | 1.78 ± 0.01 | 1.78 ± 0.01 |  |
|  |  | Hindleg | 1.9±0.01 | 1.7±0.04 | 2.03 ± 0.02 | 2.07 ± 0.01 | 0.74 ± 0.05 | 0.73 ± 0.05 | 0.73 ± 0.04 |  |
| **Mean** | **5.37±0.41** | **Foreleg** | **Not measured** | | **1.22 ± 0.12** | | **0.46 ± 0.04** | | **0.46±0.04** | **8.7±4.82** |
|  |  | **Midleg** | **2.3 ± 0.16** | | **1.92 ± 0.09** | | **1.80 ±0.06** | | **1.80±0.06** |  |
|  |  | **Hindleg** | **1.8 ± 0.07** | | **2.00 ± 0.10** | | **0.79 ± 0.06** | | **0.79±0.06** |  |

**Table S2. Morphological data of *Gerris latiabdominis*.** Morphological measurements of *Gerris latiabdominis* (n = 6) extracted from Videographic material Type 1, analyzed using Tracker software: body length (mm), leg segment lengths (femur, tibia, tarsus) (mm), maximal wetted leg length (Max. WL) (mm), and body mass (mg). For each individual, multiple observations were analyzed to calculate the **average ± SD**, resulting in a single mean morphological data set for *G. latiabdominis* based on a total of 12 observations. The foreleg femur measurements were excluded from the analysis as they were obscured by the insect’s head and antennae in the videos. Body mass was directly measured on the scale. Video clip ID indicates the name of specific video clip with the analyzed strokes in our archives.

| **IND**  **ID**  (clip IDs) | **Body length (mm)** |  | **Femur length**  **(mm)** | | **Tibia length**  **(mm)** | | **Tarsus length**  **(mm)** | | **Max. WL**  **of one leg**  **(mm)** | **Mass**  **(mg)** |
| --- | --- | --- | --- | --- | --- | --- | --- | --- | --- | --- |
|  |  |  | Left | Right | Left | Right | Left | Right |  |  |
| **1**  C0030  C0035 | 10.22±0.10 | Foreleg | - | - | 1.54±0.02 | 1.51±0.13 | 0.91 ± 0.00 | 0.91±0.05 | 0.91±03 | 19.1 |
|  |  | Midleg | 6.33±0.04 | 6.37±0.05 | 5.00±0.04 | 5.01±0.03 | 2.97 ± 0.09 | 2.94±0.17 | 7.96±0.09 |  |
|  |  | Hindleg | 5.92±0.13 | 6.02±0.06 | 3.78±0.01 | 3.71±0.04 | 2.02 ± 0.01 | 2.01±0.04 | 5.76±0.04 |  |
| **2**  C0040  C0042 | 9.85±0.02 | Foreleg | - | - | 1.44±0.07 | 1.40±0.03 | 0.78 ± 0.02 | 0.83±0.01 | 0.80±0.04 | 16.7 |
|  |  | Midleg | 6.34±0.01 | 6.39±0.03 | 4.72±0.01 | 4.77±0.01 | 3.00 ± 0.02 | 3.02±0.01 | 7.75±0.05 |  |
|  |  | Hindleg | 5.91±0.03 | 5.91±0.01 | 3.53±0.01 | 3.50±0.11 | 1.83 ± 0.01 | 1.82±0.03 | 5.34±0.08 |  |
| **3**  C0053  C0057 | 9.95±0.01 | Foreleg | - | - | 1.45±0.05 | 1.47±0.11 | 0.86 ± 0.01 | 0.90±0.01 | 0.88±0.02 | 17.7 |
|  |  | Midleg | 6.30±0.01 | 6.35±0.01 | 4.63±0.16 | 4.77±0.00 | 2.90 ± 0.02 | 2.95±0.02 | 7.62±0.13 |  |
|  |  | Hindleg | 5.91±0.01 | 5.96±0.01 | 3.59±0.02 | 3.61±0.01 | 1.93 ± 0.08 | 1.95±0.06 | 5.53±0.06 |  |
| **4**  C0061  C0062 | 10.09±0.03 | Foreleg | - | - | 1.57±0.01 | 1.55±0.01 | 0.75 ± 0.02 | 0.79±0.01 | 0.77±0.03 | 18.1 |
|  |  | Midleg | 6.28±0.01 | 6.33±0.01 | 4.76±0.04 | 4.81±0.04 | 2.89 ± 0.03 | 2.94±0.03 | 7.70±0.06 |  |
|  |  | Hindleg | 5.81±0.01 | 5.86±0.01 | 3.53±0.04 | 3.55±0.04 | 1.97 ± 0.04 | 1.98±0.03 | 5.51±0.02 |  |
| **5**  C0069  C0073 | 9.99±0.00 | Foreleg | - | - | 1.45±0.07 | 1.42±0.08 | 0.91 ± 0.00 | 0.93±0.01 | 0.92±0.01 | 15.6 |
|  |  | Midleg | 6.39±0.05 | 6.42±0.02 | 4.87±0.03 | 4.92±0.03 | 2.84 ± 0.01 | 2.89±0.01 | 7.76±0.07 |  |
|  |  | Hindleg | 5.85±0.01 | 5.90±0.01 | 3.74±0.04 | 3.75±0.04 | 1.84 ± 0.04 | 1.87±0.02 | 5.60±0.06 |  |
| **6**  C0083  C0084 | 9.88±0.03 | Foreleg | - | - | 1.46±0.10 | 1.45±0.04 | 0.76 ± 0.01 | 0.81±0.01 | 0.79±0.03 | 17.5 |
|  |  | Midleg | 6.27±0.03 | 6.32±0.03 | 4.75±0.01 | 4.79±0.01 | 2.98 ± 0.04 | 2.99±0.06 | 7.75±0.06 |  |
|  |  | Hindleg | 6.04±0.06 | 6.00±0.05 | 3.57±0.01 | 3.60±0.02 | 1.97 ± 0.02 | 1.99±0.01 | 5.56±0.04 |  |
| **Mean**  **Total: 12 clips** | **10.00 ± 0.14** | **Foreleg** | **-** | | **1.47 ± 0.07** | | **0.84 ± 0.07** | | **0.84 ± 0.07** | **17.45 ± 1.20**  **n=6** |
|  |  | **Midleg** | **6.34 ± 0.05** | | **4.81 ± 0.12** | | **2.94 ± 0.07** | | **7.76 ± 0.11** |  |
|  |  | **Hindleg** | **5.92 ± 0.08** | | **3.62 ± 0.10** | | **1.93 ± 0.08** | | **5.55 ± 0.14** |  |

**PART 2. Behavioral observations of live insects**

**Commentary to Figures S3, S4, S5:**

We observed the fans unfolding right from the start of midleg’s backward push during a typical fast stroke (Video S3 Part 2). We also observed a wide range of durations of fan opening and closing during body turning (Fig. S3B), in situations prior to the thrust phase (Video S3 Part 2) or at rest (Video S3 Part 1). The insects protracted and retracted their fans repeatedly even at static positions (at rest; Fig. S4), sometimes not to the full extent (Video S3 Part 1).

After watching 110 slow motion movies (each containing 1-4 fan opening and closing events; see examples in Video S3; Fig. S4, S5), we concluded that all our observations taken together are consistent with active control by *Rhagovelia distincta* of the time, degree and duration of expanding and closing of their fans because the initiation of unfolding or folding of the fan occurred regardless of the manner in which tarsomere 3 was applied to the water surface (Fig. S4), especially considering the angle of tarsus ventral surface to the water surface (or the distance from tarsomere2/tarsomere3 joint to the water surface, i.e., the “gap” in Fig. 1B1). While the occasional observations of fan spreading initiation at the moment when tarsomere 3 is being put down onto the water surface (Fig. S4C; i.e., when the “gap” decreases), and observations of fan closing initiation when tarsomere 3 distance to water starts increasing (Fig. S4D; i.e., when the “gap” increases) may be viewed, following Ortega-Jimenz et al. (2025), as consistent with the passive actuation, they may as well be observed with particular timing of active fan actuation coordinated with leg movement. Most crucially, however, large portion of observations are only consistent with the active fan control. For example, we observed that the tarsomere 3 position barely changed while the fan was being spread and subsequently being closed (Fig. S4A, B). We also observed that the tarsomere 3 position did not change while the fan was being unfolded up until the moment of about 80% spread, when it became engaged in pushing water while the tarsus lowered down, and fan spread to 100%. These observations collectively are inconsistent with “passive fan opening/closing”, but all of them are consistent with the active fan control through the actions of muscle(s) and elasticity in accordance with the mechanism proposed 50 years ago by Andersen (1976) and further confirmed by us in SI Part 3B.

2 mm

2 mm

2 mm

2 mm

**Figure S2. Leg application to water during a stroke by the study species.** (A) lateral view of *R. distincta* on water surface and the angle between surface and fan marked in blue. (B) Fan spread (orange) and dimple created (blue) indicate the contribution of hydrodynamic and surface tension forces to thrust. (C, D) Series of frames from a movie of a midleg stroke in *R. distincta* viewed from behind (C) and from the side (D). (E, F) Series of frames from a movie of a midleg stroke in G. latiabdominis. Blue in some frames schematically indicates the asymmetric dimple under midlegs and the posteriorly created wave bow; circular orange arrow indicates that leg rotates slightly around its longitudinal axis resulting in posterio-ventral surface of midleg pressing against the surface of water. (G, H) Comparison of cross-section of a dimple created by *R. distincta* (G) and G. *latiabdominis* (H).

**Figure S3. Fan application to water during a stroke by *R. distincta*.** (A) - the method of estimating the surface area of a fan: using ImageJ software, 6 distances between fan center and fan edge were measured, mean value was calculated and treated as a radius of a circle that includes the fan (radius of 0.85 ± 0.01 mm, n=6). The surface area of the section of the circle containing the fan indicated by the central angle, *ϴ*, was calculated using trigonometry ($Sector area=\frac{\theta\pi r^{2}}{360}$) and was used as an indicator of fan surface area (0.89 ± 0.04 mm^2^, n=6). (B) – duration of fan opening and closing for all observed instances and for selected different situations; (C, D) - examples of the angle between the plane of the surface of the fan and the water surface; (E) Distribution of angles between fan surface and water surface (mean±SD; 80.2±6.0^o^, *n*=26; *min*=65^o^, *max*=90^o^).

200 µm

1 mm

**Figure S4.** **Fan opening and closing by *Rhagovelia distincta*.** (A) – fan opening (white font) and closing (yellow font) while tarsomere 3’s position does not change as indicated by the constant angle and constant distance from water surface to the tarsomere 2/ tarsomere 3 joint marked in the frames (inconsistent with “passive mechanism” of fan opening / closing. (B) – as (A) but tarsomere 3 is closer to the water surface. (C) – fan opening associated with lowering of the tarsus onto the water surface (consistent with passive mechanism of fan opening/closing) followed by fan closing without lifting of the tarsus from the water surface (inconsistent with “passive mechanism’ of fan opening /closing). (D) – fan opening while tarsus is being lowered down towards the water surface, followed by fan closing while tarsus is being lifted upward from the water surface (consistent with the “passive mechanism” of fan opening /closing. The movie sequences can be viewed in Video S3 Part 1.

600 µm

**Figure S5. View of a stroke in *R. distincta* from under water.** A series of frame shots from a movie showing example of fan opening, thrust-generating push backwards, and fan closing viewed from below of the water surface. At rest, tarsomere 3 and distal section of tarsomere 2 create dimple on the water surface while 2-4 tips of fan’s setulae slightly expand into the water surface (from -4 to 0ms). Pushing backwards and fan expansion start at 0ms. Full length of tarsomere 2 creates a dimple during the stroke near the end of fan opening (at 11-14ms). Stroke lasts until ~65ms and fan closing lasts from ~70 to ~76ms while tarsomere 3 and 2 continue to create dimples on water surface. The movie sequence is in Video S3 Part 4.

400 µm

**PART 3. Microscopy and micro-morphology**

3A) Optical microscopy and SEM – details of methods

Using optical microscopy, we observed the morphology and behavior of the fan in specimens of Rhagovelia. Using Scanning Electron Microscopy, we visualized the structures on leg sections that interact with water. For Scanning Electron Microscopy, the specimens were stored in either 75% EtOH or 4% formaldehyde and were subjected to either a series of graded ethanol solutions (10 minutes each in 80%, 90%, 96% and subsequently 30 minutes in 100%) or a more extensive series of graded ethanol (10 minutes each in 30%, 50%, 70%, 80%, 90%, 96% and subsequently 30 minutes in 100%), respectively. Post-dehydration, all samples were gently vortexed (Vortex-Genie 2: Z258423, Scientific Industries, USA**)** in 99.9% acetone (20 minutes) for the removal of impurities and natural waxes on the body surface. Dehydrated samples were subjected to critical point dryer (CPD: SPI13200, SPI, USA) equipped with three manually operated valves, allowing control of the rate of exhaustion of carbon dioxide to minimize damage to sensitive drying artifacts such as insects (e.g. leg bending) (Bozzola & Russell, 1998). Sputter coater (automatic magnetron sputter coater: 108 auto sputter, Cressington, UK) was used to coat the dried samples with a thin layer of gold. Scanning Electron Microscopy (SEM: JSM-6390LV, JEOL, Japan) was subsequently used to produce images of hair layers on the surface insect cuticle, focusing on the water interacting sections.

Bozzola, J. J., & Russell, L. D. (1998). Electron Microscopy: Principles and Techniques for Biologists. Jones and Bartlett Publishers.

3B) Microscopic dissection and experiments with fans of dead specimens of *Rhagovelia*

The currently proposed passive control hypothesis is based on a coincidental similarity of the average fan opening duration between the artificially dissected fan in water and the fan opening duration by animals. While we confirmed this finding, we also found that such a passive elastocapillary opening does not happen in a more natural preparations of insect legs where fan as well as the claws remain attached to the tarsus at the while being disconnected from the muscle by cutting the ut tendon. Microscopic observations of midlegs of dead specimens corroborated the active rather than the passive mechanism of *Rhagovelia*’s fan extension suggested by direct observations of live insects above and confirmed by Andersen (1976).

Firstly, we established that no passive opening of the fan occurred after placing midleg tarsus ventral side on the water surface regardless of whether the leg specimen was used immediately after removing it from the ethanol solution (75%), after washing ethanol away with in water, or after drying it before the experiment. However, in some cases the tips of the 2-3 seta with setules extended into the water through the water surface (Fig. S6B-E). Similar results were observed in experiments with midleg tarsus fully pushed into the water.

Secondly, we cut the tarsomere 2 in half or near its distal end (Fig. S6C) and subsequently removed the cuticle from distal section of tarsomere 2 to expose the tendon (unguitractor tendon; *ut*) that is attached to the root of the claw+fan structure within tarsomere 3 (visible in Fig. S8C, S9B-D; confirming the original descriptions by Andersen 1976). In this preparation, the proximal end of the tendon (not visible in Fig. 6C as it is inside the leg) normally connected to the claw retractor muscle was cut off from the muscle, which then did not create tension on the *ut* because of this surgery. However, the attachment of the claw and fan to the interior walls of the basal (proximal) end of the cleft between the two lobes was not disturbed. Hence, the preparation represented natural attachment of the fan to leg structure that is not constrained by the muscle attached to *ut*. Placing this preparation on the water surface or immersing it in the water did not result in passive fan expansion. This does not support the passive expansion mechanism proposed by Ortega-Jimenez *et al.* 2025.

However, some elastocapillary interactions might have occurred sometimes in this situation between tip of fan and water because, similarly to the first experiment, a very small portion of fan surface (tips of 2-4 seta and no claw expansion observed) sometimes expanded ventrally from between the two lobes through the ventral cleft (e.g. Fig. S6B, D, E). Dorsally, the fan did not expand as it seemed to be blocked by the recurved dorsal edges of the claws (Fig. S6F, G, S7, S8). However, by making the dorsal edges of claws move away from each other (e.g. by squeezing anterio-posteriorly the very base of the cleft where claws and fan are attached to the tarsomere 3) we were able to trigger in some preparations immersed in water a similarly small partial expansion of the fan (tips of 2-3 seta) upward through dorsal cleft (Fig. S6C) because the fan was no longer blocked by the recurved edges of the claw.

Finally, in this preparation of tarsomere 3 with *ut* tendon exposed and fan+claws naturally attached to the interior wall(s) of the cleft we were able to move the fan and anterior claw downward from the cleft by pulling the “*ut*” tendon (visible in preparation cut out from the leg in Fig. S9) as described in Andersen (1976). However, the *ut* tendon always broke during the first pulling. This resulted, as already observed by Andersen (1976), in the fan and claws immediately closing (albeit sometimes not completely) into the cleft again due to the apparent torsional elasticity of the anchoring point within the cleft (marked “place of attachment” Fig. S9) clearly confirming that active action of muscles pulling the *ut* tendon is needed to fully expand the fan into the water. The nature of this suggested presence of torsional elasticity at the solid connection between the internal lobe surfaces and the fan+claw structure’s basis should be further explored in the future research, but it was already noted by Andersen (1976).

The passive fan expansion in water was only observed by us in a fan totally dissected (with or without anterior claw) from its natural position in the cleft. Similar to the recent experiments by Ortega-Jimenez *et al.* (2025), our experiments with the fan-structure that is unnaturally dis-attached from the cleft show that the fan opens in the water while it closes after lifting it from the water. Clearly, in the latter situation the surface tension of the water layer surrounding the delicate fan structure prevents fan from opening in agreement with the basic elasto-capillary principles. Therefore, the wet fan closes immediately after lifting the preparation from water. As we have not performed similar experiments in fluids other than water, we do not definitely know if immersion specifically in water or in any fluid facilitates similar passive shape changes of the entirely isolated fan. This can be easily studied experimentally in the future.

400 µm

200 µm

200 µm

200 µm

200 µm

**Figure S6. Fan in the cleft**. (A) Anterio-ventral view on tarsus with fan inside of the cleft. (B) Anterio-ventral view on tarsomere 3 with tip of fan slightly expanded from the cleft. (C) Anterior view on tarsus with tarsomere 3 cut off, and indicators of how other cuts were performed during experiments; ventral “corner”/tip of the fan comprising tips of 4 seta is expanded to water; one seta expended dorsally after experimentally unblocking the dorsal expansion of fan that is normally blocked by recurved edges of the claws visible in F, C. (D, E) Posterio-dorsal views on the two lobes with a very small portion of fan partly spread downwards and a claw visible on dorsal side of the cleft. (G, F) Dorsal SEM view on the claws with recurved edges that cover the fan dorsally blocking its spreading upwards.

100 µm

**Figure S7. Posterio-dorsal view on the dorsal surface of the tarsomere 3 with two lobes and the cleft between them.** (A) overview of the whole tarsomere 3. (B) Close-up view on the dorsal recurved edge of the posterior claw that apparently blocks the distal portion of the fan from spreading upwards. (C) Close-up view on the dorsal recurved edge of the anterior claw

**Figure S8. Fan and claws unattached from their natural position in the cleft.**

200 µm

200 µm

200 µm

(A) posterior surfaces of the fan and the claws. (B) anterior surfaces of fan and anterior claw. (C) close-up on anterior surface of posterior claw. (D) close-up on anterior surface of anterior claw. *ut* indicates the unguitractor tendon of the claw retractor muscle used in actively opening the fan by *R. distincta* as proposed by Andersen (1976).

**Figure S9. Fan and claws attached (A) and unattached (B=D) from the tarsus**. (A) tarsomere 3 with the two lobes partly removed except for very proximal sections where in the cleft the fan and claws are attached to tarsus. (B) fan unattached from the tarsus with the anterior claw broken off and *ut* tendon visible. (C, D) two claws and fan partly spread: the attachment of *ut* tendon visible. (E) close-up view on the fragment of D. Circular red arrow indicates the presumed rotation of the claw around the center (presumed attachment to the internal walls of cleft) marked by yellow circle when *ut* tendon is pulled as indicated by the red arrow.

300 µm

300 µm

200 µm

3C) Types of setae – detailed SEM images

**Figure S10. Different types of setae found on *Rhagovelia distincta*’s and *Gerris latiabdominis*’s legs interacting with water surface. (A)** Microsetae (*m*). **(B)** relatively short Macrosetae 2 (*M2*). **(C)** Grooming comb (*G*). **(D)** Cuspidate setae (*C*). **(E)** Stumped setae (*S*). **(F)** long Macrosetae 2 (*M2*). **(G)** Hook setae 1 (*H1*). **(H)** Hook setae 2 (*H2*). **(I)** Spoon setae (*Sp*). **(J)** Obtuse setae (*O*). **(K-L)** Macrosetae 1 (*M1*). **(M)** Thorn setae 1 (*T1*). **(N)** Thorn setae 2 (*T2*). **(O)** Thorn setae 3 (*T3*). **(P)** Web setae (*W*). **(Q)** Leaf-blade setae (*L*). **(R)** Leaf-like setae (*l*). **(S)** Grass-blade setae (*g*). Alphabetical references correspond to Fig. 3 and Table S3. **Black** letters indicate common setae found on both *R. distincta*’s and *G. latiabdominis*’s tarsi. **Blue** letters indicate setae found only in *R. distincta*. **Green** letters indicate setae found only in *G. latiabdominis*.

**Table S3. Morphological measurements of setae and their distribution on legs of *Rhagovelia distincta* and *Gerris latiabdominis*.** Alphabetical references (column “Ref”) correspond to panels of Figure S10 (and Fig.3). Claws and fans in *Rhagovelia* are described separately. For comparisons among the three types of legs (foreleg, midlegs and hindleg), the measurements of the microstructures were conducted on tarsus, i.e., the section that interacts with water in all legs. “W” setae were not measured due to their small size and entanglement. **Width** was the diameter measured at about 1/3^rd^ of hair length.

| **Ref.** | **Type of Seta/Microstructure** | **Basal diameter**  (µm) | **Length**  (µm) | **Width**  (µm) | **“Aspect ratio”: Basal diameter to length** | **Distribution on legs** | |
| --- | --- | --- | --- | --- | --- | --- | --- |
|  |  |  |  |  |  | *Rhagovelia distincta* | *Gerris latiabdominis* |
| **COMMON SETAE IN BOTH SPECIES**’ TARSI | | | | | | | |
| A | Microsetae  (***m***) (n = 25) | 1.37 ± 0.20 | 22.93 ± 3.66 | 1.27 ± 0.04 | 0.060 | All legs | All legs |
| B | Short macrosetae 2  (*s****M2***) (n = 15) | 2.24 ± 0.46 | 50.41 ± 11.35 | 2.16 ± 0.22 | 0.044 | All legs | All legs |
| C | Grooming comb  (***G***) (n = 7) | 5.77 ± 0.44 | 50.96 ± 3.24 | - | 0.113 | Foreleg  (above tibiotarsal joint); Midleg  (above tibiotarsal joint) | Foreleg  (above tibiotarsal joint) |
| D | Cuspidate setae  (***C***) (n = 20) | 2.32 ± 0.31 | 49.29 ± 5.90 | 1.84 ± 0.25 | 0.047 | Foreleg (posterio-dorsal) | Midleg  (ventral) |
| E | Stumped setae  (***S***) (n = 10) | 3.48 ± 0.58 | 38.43 ± 4.28 | 2.12 ± 0.08 | 0.091 | Foreleg: tibia above tibia/tarsus joint | Foreleg |
| - | Smooth setae  (***s***) (n = 10) | 1.80 ± 0.19 | 23.40 ± 2.84 | - | 0.077 | All | All |
| **SETAE PRESENT ONLY IN *Rhagovelia distincta*’s** TARSI | | | | | | | |
| F | Long macrosetae 2  (*l****M2***) (n = 15) | 5.17 ± 1.82 | 128.15 ± 40.56 | 3.68 ± 0.71 | 0.040 | Midleg (dorsal);  Hindleg (dorsal tarsomere 2, 3) | |
| G | Hook setae 1  (***H1***) (n = 5) | 1.75 ± 0.45 | 36.20 ± 0.96 | 1.69 ± 0.15 | 0.048 | Midleg (tarsomere 2, 3) | |
| H | Hook setae 2  (***H2***) (n = 5) | 1.29 ± 0.20 | 28.74 ± 3.34 | 1.21 ± 0.04 | 0.045 | Forelegs (posteroventral);  Midleg (tarsomere 3: ventral edge of proximal lobe) | |
| I | Spoon setae  (***Sp***) (n =20) | 2.15 ± 0.26 | 40.49 ± 13.69 | 8.04 ± 0.36 | 0.053 | Foreleg (ventral);  Hindleg (ventral) | |
| J | Obtuse setae  (***O***) (n = 25) | 1.75 ± 0.33 | 31.19 ± 3.75 | 1.16 ± 0.19 | 0.056 | Midleg (anterior; posterior) | |
| **SETAE PRESNT ONLY IN *Gerris latiabdominis*’s** TARSI | | | | | | | |
| K | Macrosetae 1  (*s****M1 /*** *l****M1***)  (n = 21/22) | 2.49 ± 0.40 | 41.37 ± 8.19 | 1.07 ± 0.12 | 0.060 | All | |
| L |  | 2.75 ± 0.53 | 60.88 ± 9.30 | 1.28 ± 0.12 | 0.045 | All (dorsal) | |
| M | Thorn setae 1  (***T1***) (n = 20) | 4.98 ± 1.23 | 44.29 ± 5.14 | 2.81 ± 0.16 | 0.112 | Midleg; Hindleg | |
| N | Thorn setae 2  (***T2***) (n = 20) | 4.78 ± 0.55 | 54.07 ± 4.48 | 4.61 ± 0.70  (n = 13) | 0.088 | Midleg (ventral) | |
| O | Thorn setae 3  (***T3***) (n = 16) | 5.03 ± 1.05 | 64.59 ± 8.29 | 5.06 ± 0.24 | 0.078 | Hindleg (ventral) | |
| P | Web setae (*W*) | - | - | - | - | Foreleg (ventral) | |
| Q | Leaf-blade setae  (***L***) (n = 16) | 2.55 ± 0.64 | 58.40 ± 7.19 | 4.45 ± 0.15 | 0.044 | Hindleg (ventral) | |
| R | Leaf-like setae  (***l***) (n = 3) | 1.42 ± 0.44 | 68.28 ± 3.63 | 5.00 ± 1.17 | 0.021 | Hindleg (tibiotarsal joint, especially ventral; tip) | |
| S | Grass-blade setae  (***g***) (n = 10) | 1.47 ± 0.20 | 28.69 ± 5.95 | 0.47 ± 0.06 | 0.051 | Hindleg (tip) Midleg (tip), Foreleg ventral | |

**Commentary to Figure S10 and Table S3**

After examining hundreds of SEM images representing diversity of setae on legs of the two species (only legs were examined with the focus on sections interacting with water surface), we decided to distinguish 17 types of setae: 5 types present on legs in both species, 5 types present on legs of *Rhagovelia*, and 7 types present on legs of *Gerris* (Fig. 3; Fig. S10). Obviously, properties of some setae will match intermediate characteristics between similar types in our classification, especially if the main criterion of classification is size/length, or the shape of the distal flattened shape of some setae. Examples are the three types of strong setae *T1*, *T2*, *T3* (Fig. S10 M, N, O) of increasing size and slight differences in their shape, or the two length “classes” of *M2* setae (*sM2* or *lM2*; Fig. S10 B, F), or the diverse shapes and sizes of flattened distal ends in the “spoon-like” setae class (*Sp*; Fig. S10 I). Keeping this in mind, we use our classification of types as an “ideal” reference set, which allows to compare setae, their characteristics, and their arrangements among legs and between species.

In Table S3, we present results of morphological measurements of the types of setae, and schematic representations of seta type re drawn in Fig. 3 and in Fig. S10, where the setae types are grouped into those present in both species (**black** symbols), only in *R. distincta* (**blue** symbols) and only in *G. latiabdominis* (**green** symbols). In the description below we group seta types based on their shape’s similarities in order to highlight the presence of just three major classes of setae types that are present in the microstructural arrangements relevant to locomotion, (1) the pin-like shaped (with or without bending at distal ends) micro, macro and hook setae of a very wide range of size from about 20 to about 130 µm with aspect ratio range 0.04-0.06 (Table S3), (2) thick thorn-like setae ranging from about 40 to about 70 µm with aspect ratio range 0.08-0.11, and (3) setae with flattened distal ends of a variety of shapes with length ranging from about 30 to about 70 µm and aspect ratio from 0.02 to 0.06. Several additional seta types are grouped here under “Other” class of seta types.

* Micro, macro and hook setae (Fig. S10 A, B, F, G, H, K, L)

*Nanogrooves incline towards longitudinal axis along full length of the seta*

*Microsetae,* ***m*** (Fig. S10 A), and *macrosetae 1,* ***M1****,* (Fig. S10 K, L) are characterized by their tapering, pointy and frequently bent apical tips, and nanogrooves that incline towards the longitudinal axis of the setae from the proximal base. *Microsetae,* *m*, are short with small basal diameters compared to ***M1. M1*** *setae* vary in length from shorter (K) to longer (L). Moreover, they were positioned relatively orthogonally on the insect legs, like the positioning of sensory trichobothria.

*Nanogrooves ~ parallel to longitudinal axis except distal sections where they incline to axis*

*Macrosetae 2,* ***M2***, (Fig. S10 B and F) are mostly similar in size to *M1* (except the very large M2: *“long M2*”) and were classified in respect to their tapering, pointy and bent apical tips, and nanogrooves that incline towards the longitudinal axis of the setae at the distal end (running more parallel to the axis from the proximal base to the middle). Macrosetae 2 also demonstrated considerable variations in their lengths and basal diameters, which we largely divided to *short* ***M2*** (Fig. 3B) and *long* ***M2*** (Fig. 3F) distributed differently across the insect legs.

*Nanogrooves absent (or very weak)*

Two types of hook setae ***H1*** and ***H2*** of similar basal diameters and curved apex were identified in *Rhagovelia distincta*. Hook setae 1, ***H1*** (Fig. S10G) of relatively longer lengths may have traces of 1-2 nanogrooves along their axis in the lower section of the seta. Hook setae 2, ***H2*** (Fig. S10H) of relatively shorter lengths have no visible nanogrooves.

* Thorn-like setae (Fig. S10M, N, O)

Three types of thorn-shaped setae of similar basal diameters were identified in *Gerris latiabdominis* and differentiated primarily by their length and shape, with intermediate setae between three types observed as well. *Thorn setae 1,* ***T1***, (Fig. S10M), have the shortest lengths and blunt apex and were found across the insect midlegs. Nanogrooves inclined towards the longitudinal axis of the setae from the middle to the distal end while the setae width remained seemingly unchanging until reaching the apex. *Thorn setae 2*, ***T2*** (Fig. S10N), with medium lengths and pointier apex, were further characterized by increased width (“bulk”) and subsequent tapering, with nanogrooves inclining towards the longitudinal axis of the setae from the proximal base. Thorn setae 3, ***T3*** (Fig. S10O), with longest lengths and blunt apex; nanogrooves inclined towards the longitudinal axis of the setae from the proximal base while the setae width remained seemingly unchanging until reaching the apex.

* Setae with flattened distal sections/tips (Fig. S10D, I, J, Q, R, S)

*Cuspidate setae,* ***C***, were classified based on their apical structures – flattened and sharp tips (“cusp”) that may sometimes bend in SEM images (Fig. S10D). Additionally, nanogrooves inclined towards the longitudinal axis of the setae from the middle and disappeared at the distal end while the setae width remained seemingly unchanging until reaching the apex.

*Spoon setae*, ***Sp***, (Fig. S10I) is the most diverse type. *Sp setae* were classified in respect to their flattened and enlarged apex, which were typically bent inwards (“spoon”) with the flattened surface facing the water surface. Spoon setae further demonstrated considerable variations in their conformations of varying lengths and widths. On the insect forelegs (Fig. 4C), proximal setae were of greatest lengths and narrowest “spoons”, which gradually decreased and widened, respectively, towards the distal setae. A few conspicuously enlarged spoon setae were visible at the tips. On the insect hindlegs (Fig. 4A), however, proximal setae (tarsomere 1) were of medium lengths and flattened, but not widened “spoons”, which remained unchanging to intermediate tarsomere 3. Distal setae (tarsomere 3) were of longer lengths and flattened and widened “spoons”, although narrower than the widest setae found on the insect foreleg tips. Nonetheless, nanogrooves, collectively, inclined parallel to the longitudinal axis of the setae from the proximal base and gradually disappeared towards the “spoon”.

*Obtuse setae,* ***O****,* (Fig. S10J) were classified in respect to their flattened, rectangular apex, with nanogrooves inclining parallel to the longitudinal axis of the setae from the proximal base and disappeared from the middle.

*Leaf-blade setae,* ***L***, and *leaf-like setae,* ***l***, (Fig. S10Q and R) were identified primarily from their appearances. Characteristically, leaf-blade setae were classified in respect to their flattened, curved pointy apex, with nanogrooves inclining parallel to the longitudinal axis of the setae from the proximal base. Likewise, leaf-like setae were classified in respect to their flattened, widened apex (“leaf”) attached to an elongated “stem”, with nanogrooves inclining parallel to the longitudinal axis of the setae only on the “leaf” section.

*Grass-blade setae,* ***g***, (Fig. S10S) were classified in respect to their bent, flattened apex with no visible nanogrooves. They resemble long grass-blades.

* Other setae

*Small with oval apical tips*

*Smooth setae,* ***s*** (not shown in the figure) approximately similar in lengths and basal diameters with microsetae, they were differentiated in respect to their oval apical tips and most notably the absence of visible nanogrooves. *Stumped setae,* ***S***, (Fig. S10E) were classified in respect to their oval apical tips and nanogrooves that incline towards the longitudinal axis of the setae at the distal end (running more parallel to the axis from the proximal base to the middle). Setae width remained seemingly unchanging until reaching the apex. *Grooming comb,* ***G***, (Fig. S10C) is the well know structure known to occur in Heteroptera and composed of tightly arranged short seta with stumped ends.

*Very thin long and entangled*

*Web setae,* ***W****,* (Fig. S10P) were classified in respect to their overall cobweb-like appearance. Individually however, each seta was thread-like with curvature, forming a random intertwine with one another with no visible nanogrooves.

* Brief general comparison of types of seta between species

Main text focuses only on the ventral surfaces that interact with water. In general, the legs of ripple bugs were covered with a relatively less dense hair layer (compared to *Gerris*) consisting mostly of microsetae (*m*; Fig. S10A), short and long macrosetae 2 *(sM2, lM2*, Fig. S10B, F)), and obtuse setae (*O*; Fig. S10J) on midleg tarsus. Legs of *Gerris latiabdominis* were covered with a relatively denser (compared to *Rhagovelia distincta*) layer consisting mostly of microsetae (*m*), short and long macrosetae 1 *(sM1, lM1*), and short macrosetae 2 (*sM2*). Long macrosetae 2 (*lM2*; Fig. S10F) and long macrosetae 1 (*lM1*; Fig. S10K) were sparsely distributed along the dorsal tarsus in *R. distincta* and *G. latiabdominis*, respectively (Fig. 4B). Additionally, larger setae that depending on the size may match the type T1 or T3 larger) were present along the dorsal side of Gerris’s midleg (e.g. Fig. S21B).

Additionally, *Rhagovelia distincta* had grooming combs (*G*) above the midleg tibiotarsal joint and above the foreleg tibiotarsal joint (Fig. S18) and *Gerris latiabdominis* had grooming comb on distal foreleg tibia (Fig. 5C1, Fig. S22). Moreover, smooth setae (*s*) were found irregularly in all forelegs, midlegs, and hindlegs, along with sensory sensilla and trichobothria typically on the tarsal tips. *Gerris*, specifically, had relatively densely distributed stumped setae (*S*) on their anterior forelegs (Fig. 5C2, S22).

3D) *Rhagovelia* midlegs

**Figure S11. Tarsomere 3 with anterior claw in *R. distincta*.** Anterio-ventral view on the tarsomere 3 composed of anterior and posterior lobe with a cleft between them. The image shows anterior clow and fan extruded down through the ventral cleft opening.

**Figure S12. Details of the morphology and anatomy of setae and setulae of the swimming fan in *R. distincta*.**

(A, B) – view on seta and setulae. (C) close-up view on setae. (D, E) – two examples of internal structure of setae. (F. G) close-up view on setulae. (H) setulae broken off to show hollow center (not in focus).

The orientation of board-shaped setae and setulae in the fan suggests that during a stroke they press against water with their narrow sides rather than the wide sides. This orientation can be viewed as an adaptation to withstand relatively high hydrodynamic force with little deformation because in elementary beam theory^3^ the stiffness of a beam (board) of given length in response to a force perpendicular to the beam’s width is proportional to its width and to the cube of its depth. For example, a board-like seta with a cross-section 3x8 µm (seta’s thickness x width) provide larger stiffness if it moves against water with its narrower side i.e., when seta’s thickness corresponds to beam’s width and seta’s width corresponds to beam depth in terms of elementary beam theory^3^.

**Figure S13. Details of the micro-morphology of the swimming fan in *R. distincta*.** (A, B) fan in water: distances between setulae and seta are visualized to illustrate that the fan acts as “leaky paddle”. (C, D, E) images showing the folded fan fragments where the narrower edges of seta and setulae can be measured. (F) distances between original sites where setulae are attached to the seta’s surface.

**Figure S14. Details of morphology and anatomy of the claws in *R. distincta*.** (A) claws dissected from the cleft; fan removed, posterior claw overlaps anterior claw. (B-F) cross-sections showing the internal anatomy of the fan.

**Figure S15. View on interior of the cleft after posterior lobe was removed in *R. distincta*.** (A) Overview. (B, D) closeup on dorsal part with hook setae overhanging over the dorsal cleft. (C, E) small hook setae on internal wall of the cleft. (F) distal end of anterior lobe.

**Figure S16. Details of the ventral edges of the cleft between the two lobes in *R. distincta*.** (A) two lobes of tarsomere 3, and the cleft between them after fan and claws were removed. (B) closeup view on the anterio-ventral surface of anterior lobe to illustrate rows of H1 setae on ventral side and relatively loosely distributed setae on anterior surface. (C) View similar to (B) with focus on the H2 rows on the ventral edges of the posterior lobe. (D) optical microscopy view on ventral side of the two lobes on tarsomere 3. Each small arrow in B and C indicates the basis of a setae; rows of setae marked by different color.

**Figure S17. Arrangements of setae on midleg tarsomere 2 of *R. distincta.*** (A) lateral view on the posterior side of tarsomere 2. (B) closeup on the posterio-ventral side with ventral rows of hook setae, *H1*, setae (1 row) and spoon setae, *sp* (2 rows).

**Commentary to Fig. S16, S17 – hypothetical functions of ventral setae in Fig. S16, S17**

While the presence of a dimple on the water surface under tarsus proves that the full ventral arrangement of gaps and rows of setae on tarsomeres 2 and 3 provide sufficiently high hydrophobicity to allow the formation of dimples on the water surface during a stroke, the hook setae from the very ventral edges of two lobes only partially cover the cleft leaving a narrow space of 10-20µm through which capillary action may facilitate water entering the cleft where the fan is located. If this is the case, then two processes may occur simultaneously in *Rhagovelia* at different spatial scales: surface-tension-based dimple formation at larger spatial scale (under tarsomeres 2 and 3) combined with capillary action within the narrow space of ventral cleft between lobes of tarsomere 3.

3E) *Rhagovelia* forelegs and hindlegs

**Figure S18. Arrangements of setae on foreleg tarsus of *R. distincta.*** (A) anterior view of foreleg tarsus. (B) anterior view of details of setae at the ventral side of foreleg tarsus. (C) posterior view of foreleg tarsus. (D) posterior view of details of setae at the ventral side of foreleg tarsus.

**Figure S19. Arrangements of setae on hindleg tarsus of *R. distincta.*** (A) anterio-ventral side of hindleg tarsus with three tarsomeres; the arrangement of ventral specialized setae is marked with orange shading. (B) lateral view on anterior side of hindleg tarsus; the arrangement of ventral specialized setae is marked with orange shading. (C, D) ventral closeup views on proximal (C) and distal (B) hindleg tarsus; orange-shaded *Sp* setae and pink-shaded *M2* setae are visible. (E, F) lateral anterior closeup views on proximal (C) and distal (B) hindleg tarsus. (H) tip of tarsus with ventral arrangement of spoon setae, *Sp*, marked with orange shading.

3F) *Gerris* midlegs

**Figure S20. Ventral views on the arrangements of setae on midleg tibia and tarsus of *G. latiabdominis.*** (A) Ventral side of tibia and tarsus with the prominent longitudinal gaps and rows of setae arrangement on intermediate tibia, distal tibia, and tarsus marked with purple color, and the less prominent arrangement on proximal tibia marked with blue color. (B, C) ventral views on tarsus with prominent longitudinal gaps and rows of *M1*, *C*, and *T2* setae marked with purple. (D, E) closeup views on the “gaps & rows” arrangement on ventral tarsus with different types of setae marked with different colors. (F) details of the *T2* setae.

**Figure S21. Lateral views on the arrangements of setae on midleg tarsus of *G. latiabdominis.*** Sc (A) lateral view on midleg tibia and tarsus with ventral surface marked with purple. (B) lateral posterior surface of midleg tarsus with ventral setae marked with purple shading. (C) closeup view on the anterior side of ventral tarsus with M1 and C setae marked in color.

3G) *Gerris* forelegs and hindlegs

**Figure S22. Arrangements of setae on foreleg tarsus of *G. latiabdominis.*** (A) grooming comb and stumped setae of the grooming brush on distal tibia. (B) tibia-tarsus joint covered with *W* setae and *g* setae. (C) anterio-ventral view on foreleg tarsus with arrangement of g setae running longitudinally along ventral side. (D) closeup view on *g* setae on ventral side of foreleg tarsus.

**Figure S23. Ventral structures on the hindleg of G. *latiabdominis*.** (A) ventral side of hindleg tibia with a “beam” of overlapping distal flattened ends of *L* setae marked in yellow. (B) lateral view on anterior side of hindleg tarsus with distal flattened sections of L setae marked with yellow color. (C, D) closeup view on the distal flattened sections of overlapping *L* setae that form a ventral “beam”. (E) closeup view on the T3 setae.

PART 4. Contact angles

**Figure S24. Examples of sequences of video-frames showing changes of droplet size and shape (A-E), and the results of contact angle and drop shape measurements for small droplets deposited on *R. distincta* and *Gerris latiabdominis* leg microstructures (a-e). (A, C)** Examples of changes of water droplet size and shape on the dorsal surface (air-interface) of midleg tarsus of *Gerris latiabdominis* and *Rhagovelia distincta*, respectively, through time.(**B, D)** Examples of changes of water droplet size and shape on the ventral interface of midleg tarsus of *Gerris latiabdominis* and *Rhagovelia distincta*, respectively, through time. **(E)** An example of changes of water droplet size and shape on the surface of the swimming fan’s claw in R. distincta through time. **(a, c)** Quantitative representations of visual changes (height and width) in water droplet and respective contact angles (proximal and distal) on the dorsal (air-interface) midleg tarsus of *Gerris* and *Rhagovelia*, respectively, through the relative time (100% = time when droplet disappeared). **(b, d)** Quantitative representations of visual changes (height and width) in water droplet and respective contact angles (proximal and distal) on the ventral (water-interface) midleg tarsus of *Gerris* and *Rhagovelia*, respectively, through the relative time (100% = time when droplet disappeared). **(e)** Quantitative representation of visual changes of water droplet height and width and respective contact angles (proximal and distal) of a droplet on the swimming fan claw, which normally penetrates the water body during strokes.

**Commentary to Figure S24**

The water droplet on the dorsal side of midleg tarsomere 3 of *Rhagovelia distincta* broke its sphericality soon upon landing on the hydrofuge hair layer, around 30-40% in through dissipation. Accordingly, the droplet expanded onto the insect leg successively decreasing in height and increasing in length (increased shape index). The measured contact angles, hence, systematically decreased from hydrophobicity to hydrophilicity, demonstrating relatively high wettability of the insect leg (Fig. 6A; Fig. S24C and c). The water droplet on the ventral side of the midleg tarsomere 3 of *Rhagovelia*, on the other hand, retained its semicircle conformation since landing on the hair layer only reducing in height through dissipation. The measured contact angles, respectively, remained under 90°, together demonstrating hydrophilicity of the insect leg, especially of the water-interacting side and equipped microstructures (Fig. 6B; Fig. S24D and d). The analysis of contact angle measurement in interaction with the claw that protrudes from *Rhagovelia*’s third tarsomere and penetrates the surface film of water demonstrated similar patterns to the ventral side of the insect’s midleg tarsomere 3 (Fig. 6C; Fig. S24E and e).

Contrastingly, the water droplet on the dorsal side of midleg tarsomere 2 of *Gerris latiabdominis r*etained its conformation upon landing on the hydrofuge hair layer. The droplet maintained its spherical form only reducing in size proportionately through dissipation (constant shape index: width/length ≈ 1.50). Accordingly, the measured contact angles remained within the range of 120° to 135°, together demonstrating clear hydrophobicity of insect leg (Fig. 6D; Fig. S24A and a). The water droplet on the ventral side of midleg tarsomere 2 of *Gerris*, on the other hand, while showing a similar pattern to the water droplet on the dorsal side of the leg, demonstrated reduced hydrophobicity and augmented wettability of the insect leg. Contact angles remained within the range of 100° to 120° 70% in through dissipation, after which contact angles dropped below 100° and sphericality broke (increased shape index) (Fig. 6E; Fig. S24B and b).

PART 5. Evaluation of horizontal net forces

**Figure S25. Horizontal net force profiles in slow (long) and fast (short) strokes of *R. distincta* (A) and *G. latiabdominis* (B), and tabulated summary of estimated forces (µN) in *R. distincta* (C) and *G. latiabdominis* (D).** Profiles are determined separately for fast and slow strokes separately (A, B). Summary statistics in C, D are represented for the full sample and separately for fast and slow strokes.

PART 6. Kinematics – statistical results

6A) Comparison of strokes between species

**Table S4. Statistical comparisons between species in behavioral variables characterizing a stroke.** Linear mixed model used: (Behavioral Variable) ~ species + (1 | individual). Data comprises 21 strokes of *Rhagovelia* and 12 strokes of *Gerris*) measured from 6 individuals per species. Results are shown in Fig. 8A-C. NS indicates a non-significant difference. Bold font indicates statistically significant differences. * P<0.05; ** P<0.01, *** P<0.001, NS – non-significant (P>0.10), marginally NS – marginally non-significant (0.05<P<0.1). *G. latiabdominis* is the reference level in the variable “Species”.

| Behavioral  Variable | Effects | Estimate | df | t-value | p-value |  |
| --- | --- | --- | --- | --- | --- | --- |
| Body movement | | | | | | |
| Final body velocity | (Intercept) | 300.184 | 10.317 | 7.198 | 2.48e-05 | *** |
|  | Species: *R. distincta* | -48.325 | 8.229 | -0.857 | 0.416 | NS |
| Maximum acceleration | (Intercept) | 21277.478 | 10.071 | 7.009 | 3.54e-05 | *** |
|  | Species: *R. distincta* | -5646.935 | 8.697 | -1.356 | 0.209 | NS |
| Midleg movement velocities | | | | | | |
| **Average angular velocity** | (Intercept) | 1467.44 | 15.19 | 7.398 | 2.06e-06 | *** |
|  | Species: *R. distincta* | 1011.30 | 9.58 | 3.938 | 0.00302 | ** |
| Average leg velocity | (Intercept) | 243.656 | 11.169 | 10.545 | 3.8e-07 | *** |
|  | Species: *R. distincta* | -42.507 | 6.577 | -1.417 | 0.202 | NS |
| Midleg movement distances and durations | | | | | | |
| **Midleg’s stroke amplitude** | (Intercept) | 9.7013 | 13.0447 | 17.775 | 1.59e-10 | *** |
|  | Species: *R. distincta* | -3.4634 | 9.9595 | -4.746 | 0.000794 | *** |
| **Distance traveled by wetted midleg** | (Intercept) | 9.7324 | 13.2658 | 17.838 | 1.17e-10 | *** |
|  | Species: *R. distincta* | -3.3350 | 10.2199 | -4.567 | 0.000974 | *** |
| **Stroke duration** | (Intercept) | 0.057523 | 10.116627 | 12.419 | 1.89e-07 | *** |
|  | Species: *R. distincta* | -0.018734 | 8.457357 | -2.966 | 0.0169 | * |

6B) Effect of kinematic variables on body speed

**Figure S26. Effect of leg kinematic variables on body velocity at the end of a stroke.** (A) relationship between average leg velocity (*x*-axis) and final body velocity (*y*-axis) for the two species. (B) relationship between stroke duration (*x*-axis) and final body velocity (*y*-axis) for the two species. (C) relationship between distance traveled by wetted midleg during thrust phase (*x*-axis) and final body velocity (*y*-axis) for the two species. (D) relationship between stroke duration (*x*-axis), average leg velocity (*y*-axis) and final body velocity (*color coded*) for the two species. Statistical results for A, B, C are shown in Tables S5, S7, S7. Dark blue represents faster *R. distincta* and light blue represents slower *R. distincta*; dark green represents faster *G. latiabdominis* and light green represents slower *G. latiabdominis*. Shaded areas represent 95% confidence intervals (A, B, C).

The slope of the positive linear relationship between average leg velocity and final body velocity (Figure S26A) as well as the slope of the negative relationship between stroke duration and final body velocity (Figure S26B) did not differ statistically between species (Table S5, S6). However, as the distance traveled by midlegs during a stroke decreased, the final body velocity decreased at a faster rate in G. latiabdominis than in R. distincta (Figure S26C; Table S7), resulting in faster body movement in R. distincta than G. latiabdominis for strokes of shorter distance. However, those results do not independently evaluate the effects of each variable on body speed because of correlations between the kinematic variables (e.g., Figure S26D), which cover different range values between species (shaded rectangles along *x* and *y* axes in Figure S26A, B, C). Therefore, in the main text (Table 1; Fig. 8D) the results of Principal Component Analyses based on the kinematic variables characterizing a stroke are presented.

**Table S5. Effect of leg velocity on final body velocity in a stroke in the two species.**

Results of the linear mixed model: final body velocity ~ average leg velocity + species + average leg velocity : species + (1 | individual). Data comprises 21 strokes of *Rhagovelia* and 12 strokes of *Gerris* measured from 6 individuals per species. Results are shown in Fig. S26A. The reference species is *Gerris latiabdominis*. * P<0.05; ** P<0.01, *** P<0.001, *NS* – non-significant (P>0.05).

| Effects | Estimate  ± SE | df | t-value | p-value |  |
| --- | --- | --- | --- | --- | --- |
| Intercept | 79.88±39.46 | 28.16 | -2.025 | 0.05 | *** |
| Leg velocity | 1.56±0.14 | 26.60 | 11.371 | 1.026e-11 | *** |
| Species: *R. distincta*  (*G. latiabdominis* is the reference, and the estimate concerns theoretical leg velocity = 0) | 100.68±53.49 | 28.60 | 1.882 | 0.070 | *NS* |
| Leg velocity x Species: *R. distincta* | -0.40±0.20 | 23.70 | -1.958 | 0.062 | *NS* |

**Table S6. Effect of stroke duration on final body velocity in a stroke in the two species.**

Results of the linear mixed model: final body velocity ~ stroke duration + species + stroke duration : species + (1 | individual), No. of total observations: 33 (21 *Rhagovelia distincta* and 12 *Gerris latiabdominis*), No. of individuals per species: 6. Results are shown in Fig. S26B. The reference species is *Gerris latiabdominis*. * P<0.05; ** P<0.01, *** P<0.001, *NS* – non-significant (P>0.05).

| Effects | Estimate  ± SE | df | t-value | p-value |  |
| --- | --- | --- | --- | --- | --- |
| Intercept | 738.81±61.48 | 29.0 | 12.017 | 8.76e-13 | *** |
| Stroke duration | -7.63±1.02 | 29.0 | -7.481 | 3.03e-08 | *** |
| Species: *R. distincta*  (*G. latiabdominis* is the reference,  and the estimate concerns theoretical stroke duration = 0) | -130.55±96.49 | ` | -1.353 | 0.187 | *NS* |
| Duration x Species: R. *distincta* | -1.65±2.13 | 29.0 | -0.771 | 0.447 | *NS* |

**Table S7. Linear mixed model of final body velocity and distance traveled by wetted midleg of *R. distincta* and *G. latiabdominis*.**

Results of the linear mixed model: final body velocity ~ distance traveled + species + distance traveled : species + (1 | individual). No. of total observations: 33 (21 *Rhagovelia distincta* and 12 *Gerris latiabdominis*), No. of individuals per species: 6. Results are shown in Fig. S26C. The reference species is *Gerris latiabdominis*. * P<0.05; ** P<0.01, *** P<0.001, *NS* – non-significant (P>0.05).

| Effects | Estimate  ± SE | df | t-value | p-value |  |
| --- | --- | --- | --- | --- | --- |
| Intercept | -425.85±130.39 | 26.52 | -3.266 | 0.003 | *** |
| Distance traveled | 74.60±12.79 | 23.68 | 5.833 | 5.41e-06 | *** |
| Species: *R. distincta*  (*G. latiabdominis* is the reference, and the estimate concerns theoretical distance traveled = 0) | 417.01±150.89 | 27.62 | 2.764 | 0.010 | * |
| Leg velocity x Species: *R. distincta* | -34.23±16.33 | 23.47 | -2.097 | 0.047 | * |

PART 7. Evaluation of Reynolds number and leakiness of the fan of *Rhagovelia*

7A. Evaluation of *Re*

Once the range of the Rhagovelia midleg speeds and the range of diameters of setules in the fan were collected, we set out to evaluate the value of Reynolds number (*Re*) for conditions in which the fan operates in the water, followed by estimation^4^ of fan “leakiness” based on *Re* and the typical distances between setulae in the fan. It was done for a simplified assumption that an oar is made of uniform setules (ignoring the presence of setae, assuming one specific diameter and one specific distance between setulae in an open fan). The diameters and distances between setulae considered were based on the observed values.

Reynolds number was calculated using the equation:

$$Re=\frac{\rho vL}{\mu}$$

*ρ* is the density of the fluid (water) assumed to be 1000 kg/m3, *v* is the velocity of the leg (m/s), *L* is the characteristic length (here assumed to be the diameter of a setulae comprising the fan [in meters]), and *µ* is the dynamic viscosity of the fluid, which for water was assumed to be 0.001 Pa·s (Pascal-second) equivalent to 1 centipoise (cP; representing water at 20deg C)^4^. Table S8 shows the results.

*Cengel Y.A, Cimbala J.M. 2018. Fluid mechanics fundamental and applications. 4^th^ Edition.* [*https://archive.org/details/ed4_20201119*](https://archive.org/details/ed4_20201119)

**Table S8.** Reynolds number calculated assuming *R. distincta*’s three leg velocities (0.1, 0.2 0.3 m/s) and three setule diameters (0.3, 0.5, and 0.7 $\mu$m) representing the range of empirically observed values (Fig. S12, S13 in SI part 3D). The bold underlined font indicates median value of leg speed (Fig. 8B2) and the mid-range value of the observed setule diameters, which results in ***Re*=0.1.** This value was used in estimation of fan leakiness (see below).

| **Leg velocity (m/s)** | **Setula diameter (m)** | **Estimated Reynolds number** |
| --- | --- | --- |
| **SETULAE** |  |  |
| 0.1 | 3e-007 | *0.03* |
|  | 5e-007 | 0.05 |
|  | 7e-007 | 0.07 |
| **0.2** | 3e-007 | 0.06 |
|  | **5e-007** | **0.10** |
|  | 7e-007 | 0.14 |
| 0.3 | 3e-007 | 0.09 |
|  | 5e-007 | 0.15 |
|  | 7e-007 | *0.21* |

7B. Evaluation of leakiness

Leakiness is defined as the ratio of the volume of fluid that actually moves between pair of cylinders of unit length in a unit of time to the volume between the cylinders across which they sweep in that unit of time^4^. Using Fig. 5 of Cheer & Koehl’s (1987) model of bristled appendages in arthropods that shows the estimated effect of spacing between rods of 1 or 0.1 $\mu$m diameter on the degree of leakiness of a bristled paddle made up of these rods and moving at velocities resulting in Reynolds number of *Re=*0.1, we graphically estimated the range of expected leakiness of the *Rhagovelia* fan structures composed of ~ 0.5$\mu$m diameter setules spaced at 4-10 $\mu$m gaps between neighboring setules and moving through water at 0.2m/s (which results in Re=0.1) to be in the range of 0.3-0.6 (Fig. S27).

**Figure S27. Estimation of leakiness of the swimming fan.** The theoretical relationships between the size of the gap (*x*-axis) between two cylinders/rods and the leakiness (*y*-axis) of a pair of cylinders for *Re*=0.1 and for two values of the cylinder radius: of 0.1 µm (red line) and 1 µm (blue line)^4^. The thick lines (red and blue) correspond to the range of gap values frequently observed in the fan as distances between setulae. Gray shading indicates graphically extrapolated approximate relationship expected for the cylinder radius of 0.5 µm over the range of gaps observed in the swimming fan (4-10 µm).

PART 8. Descriptions of Supplementary Videos

**Video S1. Examples of behavioral observations of *Rhagovelia distincta*.**

**Video S1. Part 1. Lateral observation of *R. distincta*’s thrust phase above water-level.**

High-speed videos showing *Rhagovelia distincta*’s locomotion during thrust phase, highlighting the interaction between wetted midleg (tarsus) and the water surface.

**Video S1. Part 2. Lateral observation of *R. distincta*’s thrust phase at water-level.**

High-speed videos showing *Rhagovelia distincta*’s locomotion during thrust phase, focusing on the protraction, use, and retraction of pretarsal swimming fan.

**Video S1. Part 3. Aerial observation of *R. distincta*’s thrust phase from directly above.**

High-speed videos capturing *Rhagovelia distincta*’s locomotion during thrust phase, illustrating the interactions between forelegs, midlegs, and hindlegs with the water surface, visualized through the shadows of the respective dimples cast below.

**Video S1. Part 4. Shadow observation of *R. distincta*’s thrust phase from directly below.**

High-speed videos showing *Rhagovelia distincta*’s locomotion during thrust phase, focusing solely on the shadows cast beneath the insect. This emphasizes the changing weight distribution on the forelegs, midlegs, and hindlegs throughout the striding motion.

**Video S1. Part 5. Velocity vectors of *R. distincta*’s thrust phase visualized using Tracker software.**

Frame-by-frame analysis of the movement of selected points on the insect’s body is represented as velocity vectors. The size and direction of the vectors illustrate the insect’s locomotion: red arrows for the body, green arrows for the midleg coxal joints, light blue arrows for the midleg tibiofemoral joints, yellow arrows for the midleg tibiotarsal joints, and pink arrows for the midleg tips.

**Video S2. Examples of behavioral observations of *Gerris latiabdominis*.**

**Video S2. Part 1. Lateral observation of *G. latiabdominis*’s thrust phase above water-level.**

High-speed videos showing *Gerris latiabdominis*’s locomotion during thrust phase, highlighting the interaction between wetted midleg (tibia-tarsus) and the water surface.

**Video S2. Part 2. Lateral observation of *G. latiabdominis*’s thrust phase at water-level.**

High-speed videos showing *Gerris latiabdominis*’s locomotion during thrust phase, focusing on the generation of asymmetrical dimple from the insect wetted midleg (tibia-tarsus).

**Video S2. Part 3. Aerial observation of *G. latiabdominis*’s thrust phase from directly above.**

High-speed videos capturing *Gerris latiabdominis*’s locomotion during thrust phase, illustrating the interactions between forelegs, midlegs, and hindlegs with the water surface, visualized through the shadows of the respective dimples cast below.

**Video S2. Part 4. Shadow observation of *G. latiabdominis*’s thrust phase from directly below.**

High-speed videos showing *Gerris latiabdominis*’s locomotion during thrust phase, focusing solely on the shadows cast beneath the insect. This emphasizes the changing weight distribution on the forelegs, midlegs, and hindlegs throughout the striding motion.

**Video S2. Part 5. Velocity vectors of *G. latiabdominis*’s thrust phase visualized using Tracker software.**

Frame-by-frame analysis of the movement of selected points on the insect’s body is represented as velocity vectors. The size and direction of the vectors illustrate the insect’s locomotion: red arrows for the body, green arrows for the midleg coxal joints, light blue arrows for the midleg tibiofemoral joints, and yellow arrows for the midleg tibiotarsal joints.

**Video S3. Examples of behavioral observations of *Rhagovelia distincta* and swimming fan use.**

**Video S3. Part 1.** Observational evidence that *Rhagovelia distincta* deliberately protracts and retracts its pretarsal swimming fan manually and purposefully.

**Video S3. Part 2.** Observational evidence that *Rhagovelia distincta* protracts its pretarsal swimming fan before striding, indicating that the swimming fan use is an active process not solely driven by water resistance.

**Video S3. Part 3.** Observational evidence that *Rhagovelia distincta* retracts its pretarsal swimming fan after striding while stationary, further demonstrating that fan is an active process independent of external water pressure.

**Video S3. Part 4.** Observational evidence showing that *Rhagovelia distincta* generates a shallow dimple along the wetted midleg during locomotion.

**Video S3. Part 5.** Observational evidence that *Rhagovelia distincta* lifts the water surface at the wetted midleg tip after striding, suggesting active manipulation of the water surface.

**Video S3. Part 6.** Observational evidence that *Rhagovelia distincta* generates a shallow dimple along the wetted midleg during a backward stroke. The dimple formation behind the pretarsal swimming fan suggests it is likely a byproduct of the drag created by the fan just below the water surface. The swimming fan adopts a “parachute shape” – bent posteriorly in a bowl-like fashion.

**Video S4. Examples of contact angle measurement.**

Contact angle measurement on *Rhagovelia distincta*’s pretarsal swimming fan blade – water droplet progressively expanding on the surface of the blade, with its proximal and distal angles measured and represented in red.

**PART 9. References**

1. Ortega-Jimenez, V., Bhamla, S., Kim, D. *et al.* Ultrafast elastocapillary fans control agile maneuvering in ripple bugs and robots. *Science* **389**, 811–817 (2025).
2. Andersen, N. M. A comparative study of locomotion on the water surface in semiaquatic bugs (Insecta, Hemiptera, Gerromorpha). *Vidensk. Medd. Dan. Naturhist. Foren.* **139**, 337–396 (1976).
3. Bhavikatti, S. S. *Mechanics of Solids*. (New Age International (P) Ltd., Publishers, 2010).
4. Cheer, A. Y. & Koehl, M. A. R. Paddles and rakes: fluid flow through bristled appendages of small organisms. *J. Theor. Biol.* **129**, 17–39 (1987).
